# Aerosolizable Lipid-Nanovesicles Encapsulating Voriconazole Effectively Permeate Pulmonary Barriers and Target Lung Cells

**DOI:** 10.3389/fphar.2021.734913

**Published:** 2022-03-10

**Authors:** Ranjot Kaur, Sarah R Dennison, Shivaprakash M Rudramurthy, O P Katare, Teenu Sharma, Bhupinder Singh, Kamalinder K Singh

**Affiliations:** ^1^ University Institute of Pharmaceutical Sciences, UGC Centre of Advanced Studies, Panjab University, Chandigarh, India; ^2^ University of Central Lancashire, Preston, United Kingdom; ^3^ Postgraduate Institute of Medical Education and Research, Chandigarh, India; ^4^ UGC Center for Excellence in Nano-Biomedical Applications, Panjab University, Chandigarh, India; ^5^ UCLan Research Centre for Smart Materials, University of Central Lancashire, Preston, United Kingdom; ^6^ UCLan Research Centre for Translational Biosciences and Behaviour, University of Central Lancashire, Preston, United Kingdom

**Keywords:** lipid nanovesicles, antifungal, pulmonary, *Aspergillus*, inhalation, nebulisation, lung targeting

## Abstract

The entire world has recently been witnessing an unprecedented upsurge in microbial lung infections. The major challenge encountered in treating the same is to ensure the optimum drug availability at the infected site. Aerosolization of antimicrobials, in this regard, has shown immense potential owing to their localized and targeted effect. Efforts, therefore, have been undertaken to systematically develop lung-phosphatidylcholine-based lipid nanovesicles of voriconazole for potential management of the superinfections like aspergillosis. LNVs, prepared by thin-film hydration method, exhibited a globule size of 145.4 ± 19.5 nm, polydispersity index of 0.154 ± 0.104 and entrapment efficiency of 71.4 ± 2.2% with improved *in vitro* antifungal activity. Aerodynamic studies revealed a microdroplet size of ≤5 μm, thereby unraveling its promise to target the physical barrier of lungs effectively. The surface-active potential of LNVs, demonstrated through Langmuir-Blodgett troughs, indicated their ability to overcome the biochemical pulmonary surfactant monolayer barrier, while the safety and uptake studies on airway-epithelial cells signified their immense potential to permeate the cellular barrier of lungs. The pharmacokinetic studies showed marked improvement in the retention profile of voriconazole in lungs following LNVs nebulization compared to pristine voriconazole. Overall, LNVs proved to be safe and effective delivery systems, delineating their distinct potential to efficiently target the respiratory fungal infections.

## Introduction

Of late, the impact of microbial lung infections on the healthcare sector has been overwhelming. The devastating Covid-19 wave, being witnessed across the world, has further augmented the susceptibility of patients to superinfections like invasive pulmonary aspergillosis (IPA) and mucormycosis (black fungus disease), thus making the treatment of the deadly coronavirus (SARS-CoV-2) even more complicated (Machado et al., 2021; Marr et al., 2021). The virus, in fact, directly targets and disrupts the airway barriers and enables the infiltration of *Aspergillus* into the lung epithelial cells, thereby causing Covid-19 associated pulmonary aspergillosis (CAPA) (Koehler et al., 2020a; Arastehfar et al., 2020). Several clinical studies have reported the emergence of CAPA, as it is associated with high mortality rates (Alanio et al., 2020; Koehler et al., 2020b; Rutsaert et al., 2020). The first-line treatment regimen includes using azoles, like voriconazole, and other antiviral agents, while liposomal amphotericin B is employed in azole-resistant cases (Koehler et al., 2020a). However, voriconazole is administered through oral and parenteral routes only, thus limiting the availability of optimum drug concentrations in the lungs for longer durations. Moreover, hepatotoxicity, a commonly observed adverse effect with the conventional therapy of voriconazole, worsens the condition of patients further (Den Hollander et al., 2006; Sharma et al., 2021). Researchers worldwide have been working hard to deal with this intricate situation and explore diverse approaches to develop a safe, effective and biocompatible formulation with minimal side effects.

As an alternative, direct delivery of antifungals to lungs has been gaining immense interest in the scientific world, as it has numerous merits of larger surface area, faster onset, thin epithelial membrane, avoidance of first-pass effect, non-invasive administration, dose reduction and high drug amounts at the targeted site (Zhou et al., 2015; Kaur et al., 2019). Although several clinical and literature instances have reported the inhalation of antifungals, availability of insufficient evidence on lung safety and efficacy tends to limit their federal approval (Rijnders et al., 2008; Hilberg et al., 2012; Andersen et al., 2017). Thus, extensive research efforts and improved drug delivery strategies are required to substantiate the role of inhalation delivery in microbial lung infections, a domain hitherto not much explored.

Till date, a wide diversity of delivery systems has been successfully employed for inhalation therapy. These encompass cyclodextrin complexes (Evrard et al., 2004), nanomicelles (Vadakkan et al., 2015), large porous particles (Garcia-Contreras et al., 2007), liposomes (Cipolla et al., 2013), nanostructured lipidic carriers (Pastor et al., 2019), solid lipid nanoparticles (Videira et al., 2012), polymeric nanoparticles (Sinha et al., 2013), and nanoemulsions (Li et al., 2016). Phospholipid-based vesicles or liposomes, in this regard, have been the most widely explored and accepted systems, as these tend to exhibit chemical similarity with the lung surfactant, i.e., 80% phospholipids, with the major fraction being dipalmitoylphosphatidylcholine (DPPC) (Elhissi, 2017). In this regard, the first lipid vesicle-based product, viz., Alveofact® (purified bovine surfactant), was introduced for pulmonary drug delivery in the 1990s, in the therapeutic management of acute respiratory distress syndrome in infants (Paranjpe and Muller-Goymann, 2014). Such drug carrier systems, enriched in phospholipids and cholesterol, offer distinct advantages of being nontoxic, biocompatible and biodegradable (Wauthoz and Amighi, 2014). Further, these have been documented to possess the ability to entrap both hydrophilic and lipophilic drug moieties, owing to the presence of an aqueous core and phospholipid membrane, respectively (Bozzuto and Molinari, 2015). Though the size of these delivery vehicles can vary from microns to nanometers, their nanometric versions are particularly preferred owing to their superior biopharmaceutical potential, majorly attributable to their significantly higher surface-area-to-volume ratio (Paranjpe and Muller-Goymann, 2014).

Several methods have been reported in literature for the preparation of liposomes, including thin film hydration, reverse-phase evaporation, solvent-injection and detergent-depletion (Bozzuto and Molinari, 2015). However, the most commonly employed laboratory technique is thin film hydration, as it is the simplest-known approach, wherein the dried lipid film is dispersed in an aqueous medium, followed by sonication or extrusion (Zhang, 2017; Xiang and Cao, 2021). Such drug delivery vehicles can be employed for the delivery of wide-ranging therapeutic drug moieties, proteins and nucleic acids through various routes of administration, like oral, topical, parentral, inhalation and nasal. (Xiang and Cao, 2021). These drug delivery systems are, in fact, the first to be successfully translated into real-time drug products for clinical applications, such as Doxil®(1995), Amphotec®(1996) and Ambisome® (1997) (Bulbake et al., 2017). Another significant factor that should be considered while developing inhalation therapies, is the mode of its delivery and the severity of the underlying illness. In severe CAPA, majority of the patients are on mechanical ventilation to support their normal breathing (Brunaugh et al., 2020). Nebulizers, in such cases, are the most preferred devices to deliver aerosolized drugs to the lungs, as these devices demonstrate the ability to deliver larger volumes of medication to the lungs compared to the corresponding pressurized metered inhalers and dry powder inhalers (Elhissi, 2017). Nevertheless, the high-shear forces involved in the process of generating respirable microdroplets (i.e., 1–5 µm), unfortunately, tend to exert physical stress and cause membrane instability, thereby causing the consequent loss of the entrapped drug molecule (Elhissi, 2017). This drug loss could be minimised by reducing the size of formulation to 1 μm or less, prior to jet nebulization (Taylor et al., 1990), or by addition of membrane stabilizers like cholesterol (Rudokas et al., 2016), or incorporating high-phase transition phospholipids like hydrogenated soya-phosphatidylcholine (HSPC) in the formulation (Niven and Schreier, 1990; Rudokas et al., 2016).

Of late, application of Quality-by-Design (QbD) principles has been increasingly permeating in the industrial practice as well as in academic environs (Singh et al., 2011). QbD is verily a rational, systematic, risk- and science-based, and resource-efficacious approach, which provides an all-inclusive know-how of varied critical process parameters (CPPs) and critical material attributes (CMAs), influencing the corresponding critical quality attributes (CQAs) of the drug product(s). Also, it assures the pharmaceutical quality and robustness of the final optimum product, well within the regulatory framework of design space (Singh et al., 2005; Sharma et al., 2020).

The present research studies were accordingly embarked upon to repurpose voriconazole for inhalation therapy with a goal to effectively target respiratory airways and improve upon its therapeutic efficacy. The drug was encapsulated into biomimetic phospholipid vesicles (LNVs), and the formulation was systematically optimized following the principles of QbD. Further, the optimized formulation was investigated in terms of their vesicle formation, physiochemical characteristics, aerosol performance, interaction with air-lung interface lipid monolayers, antifungal efficacy, safety and uptake employing broncho-alveolar epithelial cell lines, and postulation of their internalization mechanism using flow cytometer. The *in vivo* deposition and retention potential was also substantiated in Balb/c mice using a previously validated in-house nose-only inhalation chamber.

## Materials and Methods

Voriconazole was obtained *ex-gratis* by M/s LifeCare Innovations, India. DPPC and HSPC were received *ex gratis* from M/s Lipoid, Germany. Cholesterol (M/s Across, India), acetonitrile (ACN) and chloroform (M/s Fisher, India), and Rhodamine 123 (Rh-123, M/s Sigma Aldrich, United Kingdom), were purchased from the corresponding sources. All other solvents and chemicals employed during the said research work were of standard analytical grade. Lung epithelial cell lines, *viz.*, human bronchial epithelial (i.e., Calu 3) and adenocarcinoma human alveolar epithelial (i.e., A549) cells, were procured and maintained according to the methods recommended by M/s ATCC, United States.

### Method of Preparation

LNVs were formulated by thin-film hydration (Zhang, 2017) by first dissolving DPPC, HSPC and cholesterol in chloroform (10 ml) to form a clear homogeneous mixture, followed by formation of lipid-film by removing the organic solvent through rotary evaporation (R-215, M/s Buchi, Switzerland). Lipid film was dried by placing the flask overnight in a fume-hood to remove any residual solvent, and film hydration was accomplished by adding distilled water (10 ml) at 60°C and agitating. Finally, large vesicles were probe-sonicated (M/s Vibra Cell Sonics, United States) and filtered (0.22 µm filters). The LNVs loaded with voriconazole (20 mg) and Rh-123 (10 μg.ml^−1^) were also prepared analogously, involving lipid film formation and hydration.

### Formulation Development Employing Design of Experiments

A 5-variables-8-runs Fractional Factorial Design (FFD) (Supplementary Table S1 **),** was used to conduct the factor screening studies, followed by factor optimization studies employing a 3-factors-17-runs Box-Behnken Design (BBD) (Supplementary Table S2), both employing Design Expert® software 11.0 (M/s Stat-Ease, Minneapolis, United States). The prepared formulations were experimentally assessed for various CQAs like particle size (PS), zeta potential (ZP), polydispersity index (PDI), entrapment efficiency (EE) and cumulative drug release at 8 h (DR_8h_). Data analysis was accomplished employing appropriate mathematical models, followed by delineating the design space using apt numerical and graphical optimization tools (Singh et al., 2011).

### Characterization Studies

#### Size and Zeta Potential Analysis

The globule size as well as zeta potential of LNVs were estimated employing laser diffraction equipment (Nano ZS, M/s Malvern, United Kingdom) at 25°C. The formulations were diluted 50-folds using distilled water and PBS, before subjecting these to globule size analysis.

#### Entrapment Efficiency

Entrapment efficiency was determined using the centrifugation method. LNVs (i.e., 2 ml) were centrifuged at 20,000 g for 2 h at 10°C. The supernatant was analysed for free voriconazole content using HPLC technique (Kaur et al., 2021a; 2021b) with the mobile phase of acetic acid solution and ACN (50:50) at 1 ml min^−1^ of flow rate and 20 µL of injection volume. The following equation was used for the calculation of entrapment efficiency.

$$\%\;\text{Entrapment}\;\text{Efficiency}\frac{\left(\text{Total}\;\text{drug}\;\text{content}-\text{drug}\;\text{in}\;\text{supernatant}\right)}{\left(\text{Total}\;\text{drug}\;\text{content}\right)}\times 100$$

#### Field Emission Scanning Electron Microscopy

Surface morphological topographies of LNVs, drug-loaded as well as blank, were observed using FESEM (Su8010, M/s Hitachi, Chiyoda, Japan). The samples were placed over aluminium stubs and dried under vacuum, followed by their microscopic analysis. The particle size of the nanovesicles was also measured using ImageJ software (Abràmoff et al., 2004; Mazzoli and Favoni, 2012).

#### Fourier Transform Infrared Spectroscopy

The likelihood of any physicochemical interaction(s) between voriconazole and excipient(s) can be assesed by comparing it with the normal FTIR spectrum, i.e., presence (or absence) of principal peak(s) of the drug corresponding to a particular functional group. Studies were performed by KBr pellet method employing FTIR spectrometer (M/s Perkin Elmer, Massachusetts, United States). Spectra of voriconazole, HSPC, DPPC, cholesterol, physical mixture (P-mix) and of LNVs were analysed for any significant shifting of the recorded peak(s).

#### Powder X Ray Diffraction Studies

The crystallographic structure of voriconazole, P-mix and LNVs was recorded using P-XRD Model X’pert PRO (M/s PANalytical, Almelo, Netherlands). The samples were placed in round-shaped holders and were analysed using Cu radiation at 45 Kv and 40 mA. The samples were measured from 3.5 to 40° in 2θ using solid detector.

#### *In vitro* Drug Release Studies

The release profiles of voriconazole suspension and drug LNVs dispersion were obtained using the dialysis sac approach (Arora et al., 2015). Briefly, LNVs (containing 1.7 mg drug) and drug suspension, were kept in a dialysis membrane (12 kDa, M/s Sigma Aldrich, United Kingdom) with 20 ml of dissolution medium, *viz.,* phosphate buffer saline (pH 7.4) comprising of 0.1% Tween 80, at 37 ± 0.5°C and 100 rpm. Aliquots of samples of 1 ml each were withdrawn at the periodic pre-determined intervals and replaced using fresh buffer medium. Samples were subsequently subjected to drug analysis employing HPLC, and the resultant drug release data were subjected to fitting with various mathematical models to unravel the underlying release behaviour (Ahuja et al., 2007).

#### Determination of Microdroplet Size Using Laser Diffraction

Aerosol droplet size of the optimized LNVs dispersion was determined using Spraytec laser diffractometer (M/s Malvern Panalytical, United Kingdom). Performance of different types of nebulizers, *viz.,* Pari LC, Omron C803, Sidestream and Aeroneb solo, was compared on the basis of volume mean diameter (VMD), fine particle fraction (FPF) and geometric standard deviation (GSD). LNVs (10 ml) were loaded onto each nebulizer, placed perpendicular to the laser beam of Spraytec at a 3-cm distance with analysis time of 60 s (Nasr et al., 2012). A lens of 300 mm focal length was employed, covering the droplet range of 0.1–900 µm. The nebulizer yielding the best-aerosolized fraction was selected for further extensive *in vitro* and *in vivo* characterization.

#### *In vitro* Aerosol Deposition Using Next-Generation Impactor

Aerosol deposition studies on LNVs were conducted using an NGI (M/s Copley Scientific, United Kingdom) (Abdelrahim and Chrystyn, 2009). Nebulizer cup was filled with formulation (10 ml) and was coupled to the induction port of impactor using a mouthpiece adapter at the flow rate of 15 L min^−1^ employing a flow meter (DFM 2000; M/s Copley Scientific, United Kingdom). After 5 min of nebulization, each cup-holder tray was disconnected, samples were extracted with methanol (10 ml) and analyzed employing HPLC. Values of mass median aerodynamic diameter (MMAD) and GSD were determined using online software (MMAD Calculator, 2020). Emitted dose (ED), i.e., the dose available for inhalation to the total dose, and FPF, *i.e.,* mass of particles <5 µm divided by the ED, were also calculated.

#### Stability of Lipid Nanovesicles After Nebulization

Effect of nebulization on the globule size of LNVs was determined using dynamic light scattering, in triplicate, by recording particle size of LNVs placed in the nebulization cup, before and after nebulization period of 20 min (Mainelis et al., 2013).

#### Surface Activity Experiments

Langmuir Teflon trough (80 ml), equipped with movable barriers, was used to study the surface-active potential of voriconazole (1 x PBS 7.4) and its LNVs dispersion. The trough was filled with PBS 7.4 (10 mM) and adjusted to 15-cm^2^ area. The drug solution or LNVs dispersion (50 µL) was injected into the buffer sub-phase to get final concentrations of 0–100 µM using a Hamilton microsyringe. Changes in surface pressure were recorded with the Whilemly plate, coupled with a microbalance, and were plotted against respective drug concentrations (Dennison et al., 2012).

##### Lipid-Monolayer Interaction Studies

The monolayer interaction studies were conducted employing a synthetic lipid (i.e., DPPC) and natural lipids, as extracted from A549 cells (Bittame et al., 2016). Briefly, the cells were grown till 100% confluency, extracted by trypsinization, and centrifuged (350 × g) to obtain a cell pellet, which was resuspended in chloroform: methanol mixture (1:2 v/v, 3 ml), followed by vortex-mixing (30 min). The solvent mixture (500 µL) was added to purified water (900 µL), and then centrifuged at 350 × *g* (10 min) for lipid purification. The extracted lipids were further stored at −20°C after removing the residual solvents using nitrogen flux.

An approximate pressure of 30 mN m^−1^, mimicking biological membranes, was used to investigate the interaction of voriconazole and LNVs with lipid monolayers. DPPC and extracted A549 lipids (0.5 mM) were dissolved in chloroform and spread onto the buffer subphase. The trough was kept undisturbed for 30 min to evaporate the solvent. Then, a 10 cm^2^ min^−1^ velocity was applied to compress the monolayer to obtain the desired pressure. Finally, samples were individually injected into the subphase to obtain the final drug levels of 75 μM, and surface pressure changes were monitored employing NIMA software (Dennison et al., 2012).

#### Cell Culture Studies

##### Cellular Safety Studies

Studies on cell viability were conducted to investigate safety potential of the developed LNVs on lung epithelial cells. A549 (5 ×10^3^ cells/cm^2^) and Calu 3 (1 × 10^4^cells/cm^2^) cells were seeded in 96-well plates (M/s Thermo Fischer Scientific, Denmark). Following 48 h of incubation, the cells were refilled with 90 µL of fresh medium containing the optimized formulation (3.57–114 μM) and 10 µL of PrestoBlue^TM^, with 1 h of incubation period. Fluorescence was measured employing a microplate reader (M/s Tecan, Switzerland) at the excitation and emission wavelengths of 535 and 612 nm, respectively. (Khurana et al., 2017).

##### Cellular Uptake Studies

###### Qualitative Studies

A549 (1 × 10^5^ cells/well) and Calu 3 (2 × 10^5^ cells/well) cells were seeded on sterile coverslips in 6-well plates and incubated at 37°C and 5% CO_2_, till 85–90% confluency was attained. Following incubation, the culture plates were removed, cells were washed with PBS 7.4 and again incubated for 4 h with a medium containing Rh-123 LNVs (10 μg ml^−1^). Further, cells were washed thrice with PBS, fixed with 4% of paraformaldehyde and stained with DAPI, before conducting their fluorescence microscopy (M/s Carl Zeiss, United Kingdom) (Khurana et al., 2017).

###### Quantitative Studies

Time- and concentration-dependent cellular uptake studies were carried out employing the flow cytometer (Guava® easyCyte HT, M/s Merck, Germany). Different concentrations, *i.e.,* 2.5, 5 and 10 μg ml^−1^ of Rh-123 LNVs were incubated at 37 ± 1°C and 5% CO_2_ with A549 (5 × 10^4^ cells/well) and Calu 3 (1 × 10^5^ cells/well) cells in 12-well plates for various time intervals, i.e., 0.5, 1, 2, 4 and 6 h, to estimate the mean fluorescence intensity (Jiang et al., 2013; Khurana et al., 2017).

###### Mechanistic Endocytosis Pathway Analysis

Lung epithelial cells were seeded and grown in 12 well-plates till 80–90% confluency. The medium was then replaced with medium containing different endocytosis inhibitors, *viz.,* sucrose (i.e., 0.45 M), cytochalasin B (i.e., 5 μg ml^−1^) and nystatin (i.e., 5 μg ml^−1^) for selectively blocking the clathrin, caveolae/lipid rafts and macropinocytosis/phagocytosis pathways. After incubating for 90 min, the medium comprising of said inhibitors was removed, replenished with the medium containing 5 μg ml^−1^ of Rh-123 LNVs and endocytic inhibitors, and further incubated for 4 h at 37°C and 5% CO_2_. Also, energy-dependent endocytosis was conducted by incubating the cells at 4°C. After incubation, the treated samples were processed and subjected to flow cytometry for further analysis (Khurana et al., 2018).

#### Antifungal Efficacy

Antifungal testing of the optimized LNVs and VRC was determined at drug concentrations ranging between 0.029 and 14.84 μg ml^−1^, against susceptible fungal strains, *viz., Aspergillus flavus, Aspergillus fumigatus, Candida krusei* and *Candida parapsilosis,* by the microbroth dilution method^42^. The minimum inhibitory concentration (MIC) values were recorded, based upon visual end-point, endorsed by CLSI M27-A3 protocol (CLSI, 2008).

#### Plasma and Lung Pharmacokinetic Studies

All the animal experiments were conducted following ethical approval from Panjab University Institutional Animal Ethics Committee, India (PU/45/99/CPSEA/IAEC/2019/243). The animals, Balb/c mice (23.0 ± 2.0 g), were divided into two groups. Group I received voriconazole nebulization (Vorier, M/s Zydus Cadila, India), while Group II received LNVs nebulization (containing 15 mg of voriconazole) (Tolman et al., 2009). Mice were acclimatized with twice-daily nebulization of 20 min using a lab-scale nose-only inhalation chamber, already reported by us (Kaur et al., 2020). Animals were sacrificed by cardiac puncture at varied time-points, *viz.,* 0.2, 0.5, 1,2, 4, 8, 12 and 24 h (*n* = 3), each after 20 min of nebulization. Whole blood was collected in heparinized vials, and plasma was obtained by centrifugation at 9,055 × g for 10 min. Also, lungs were removed, homogenized and stored at −20°C, until sample analysis, for calculating various pharmacokinetic parameters employing PK solver software (Zhang et al., 2010).

### Statistical Analysis

Statistical analysis was conducted using Prism Software (M/s GraphPad Inc., La Jolla, United States) employing one-way analysis of variance (ANOVA) and unpaired Student’s t-test. All the experiments were carried out in triplicate.

## Results

### Formulation Development Employing Design of Experiments

Screening studies aided in selecting “*vital few influential”* input variables among “*plausible so many*”. Examination of Pareto and half-normal plots (Supplementary Figure S1) revealed statistically significant (*p* < 0.05 to 0.001) effect of variables like DPPC, HSPC and cholesterol, much above the Bonferroni and t-value threshold lines. First-order model (Eq. 1 **)** was used for computing polymer coefficients as,

$$y=\beta_{0}+\beta_{1}X_{1}+\beta_{2}X_{2}+\beta_{3}X_{3}+\beta_{4}X_{4}+\beta_{5}X_{5}\ldots$$ (1)

where, y is a specific CQA, β_0_ is intercept, and β1 to β5 are coefficients of respective linear model terms.

Optimization data analysis, employing a BBD, revealed the prevalance of distinct interaction(s) among the studied CPPs (Supplementary Figure S2 **).** The optimized formulation composition, *i.e.,* HPSC (97 mg), DPPC (56 mg) and cholesterol (19 mg), was found to be well-within the design space (Supplementary Figure S3 **).** Probe sonication time and temperature were kept constant at the values of 120 s and 60°C during all the experimental runs.

### Characterization Studies

#### Average Particle Size, Zeta Potential and Entrapment Efficiency

LNVs diluted with water had a mean diameter, PDI and ZP of 145.4 ± 19.5 nm, 0.154 ± 0.104 and 6.23 ± 1.21, respectively, while those diluted with PBS had 214.0 ± 21.5 nm, 0.259 ± 0.05 and 4.44 ± 0.14, respectively (Table 1, Supplementary Figure S4). The optimized LNVs formulation exhibited the EE values of 71.4 ± 2.2%.

**TABLE 1:** Particle size distribution of lipid nanovesicles of voriconazole (*n* = 3).

**Lipid nanovesicles**	**Particle size (nm)**	**Polydispersity index**	**Zeta potential**	**Particle size (nm)**	**Polydispersity index**	**Zeta potential**
	**Water**			**PBS**		
Before sonication	2,678.5 ± 409.5	0.508 ± 0.388	5.07 ± 0.44	3,605.1 ± 254.7	0.579 ± 0.237	3.75 ± 0.61
After probe sonication (120 s)	175.2 ± 28.4	0.427 ± 0.113	6.60 ± 0.57	233.6 ± 25.2	0.239 ± 0.165	4.46 ± 0.92
After filtration (0.22 µm)	145.4 ± 19.5	0.154 ± 0.104	6.23 ± 1.21	214.0 ± 21.5	0.259 ± 0.055	4.44 ± 0.14

#### Electron Microscopic Studies

**FIGURE 1:**
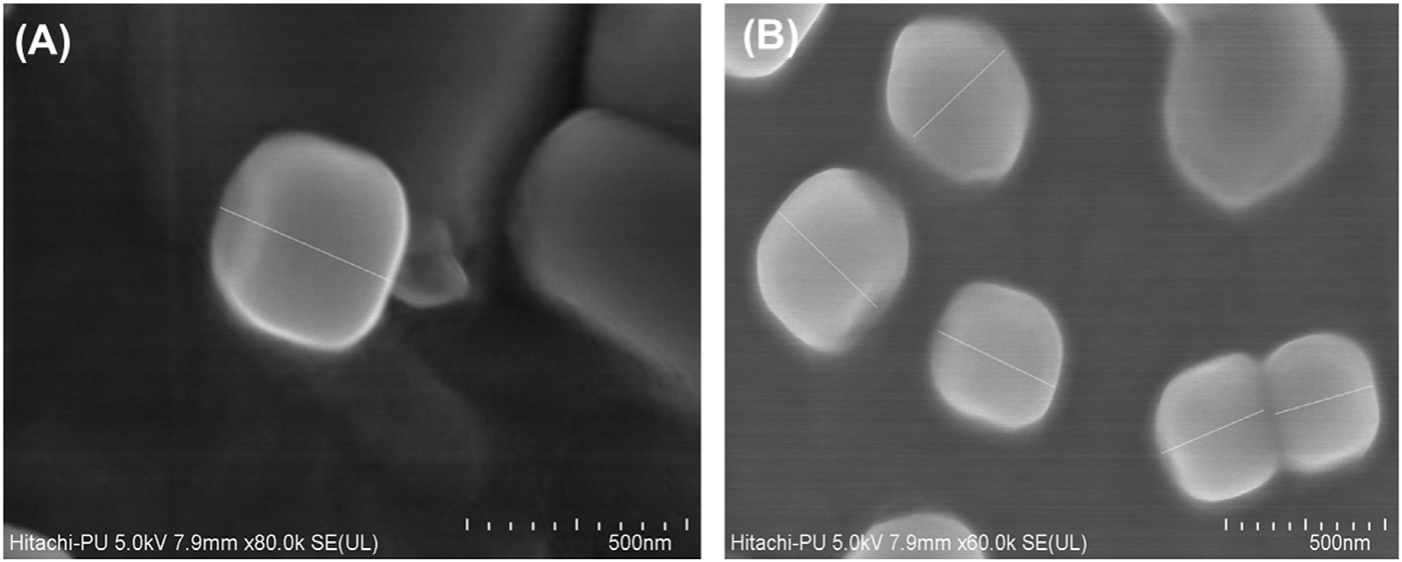
Representative FESEM images of blank **(A)** and voriconazole-loaded nanovesicles **(B)**. The scale bar indicates 500 nm with a mean (±SD) size of 248.03 (±49.16) nm, calculated using ImageJ software.

The FESEM images ratified the nanometric characteristics of the prepared LNVs (Figure 1), with slightly polygonal shape and smooth appearance. Besides, the particle size of LNVs, calculated using ImageJ software, was observed to be 248.03 ± 49.16 nm.

#### FTIR Studies

**FIGURE 2:**
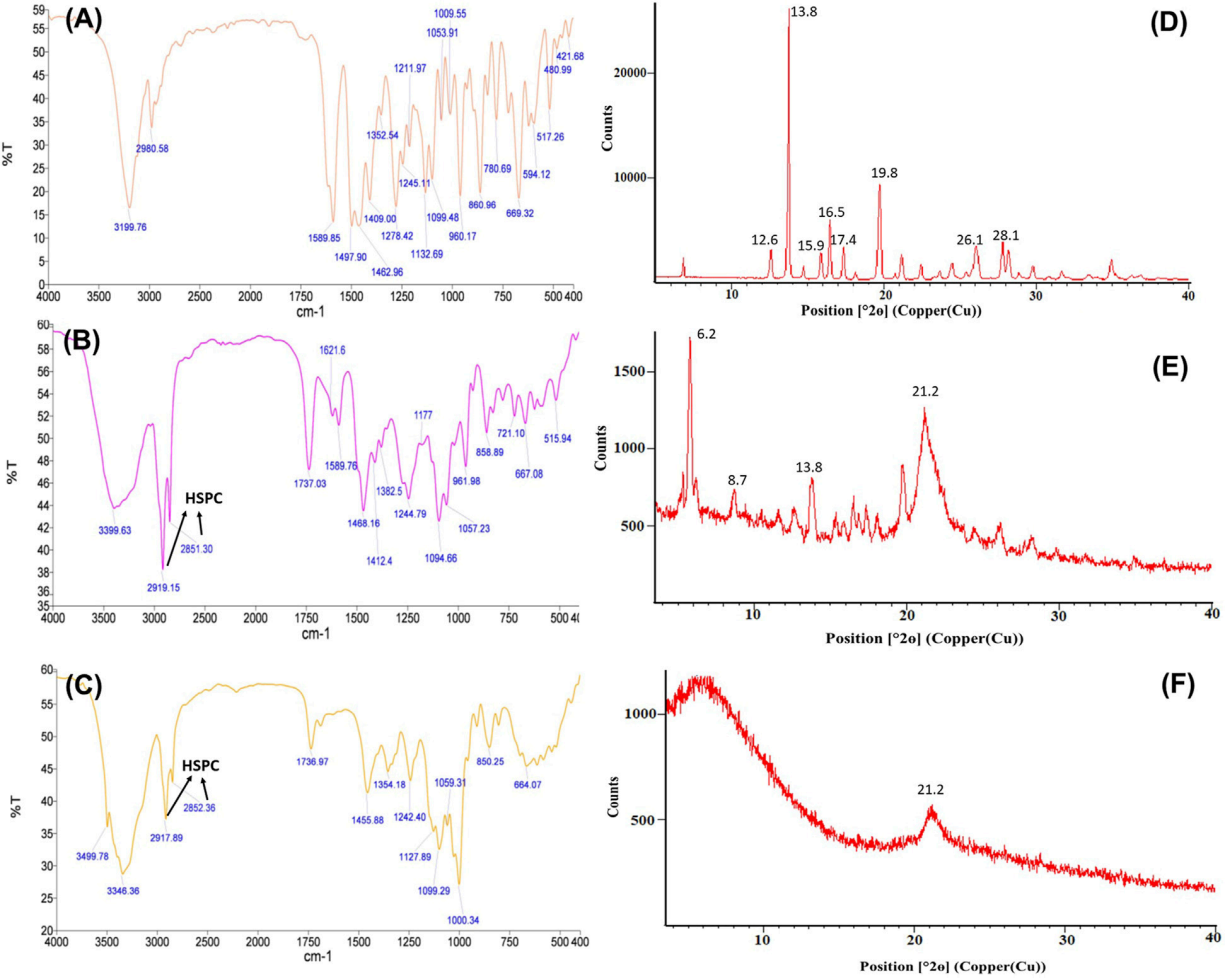
FTIR spectra of voriconazole **(A)**, physical mixture containing voriconazole, hydrogenatedsoyaphosphatidylcholine, dipalmitoylphosphatidylcholine and cholesterol **(B)**, and optimized lipid nanovesicles **(C)**. The arrows indicate the respective peaks of HSPC in FTIR spectra. The corresponding PXRD diffractograms illustrate the crystalline nature of drug molecule **(D)** and physical mixture **(E)**, compared to the amorphous state of LNVs **(F)**, delineating the respective vital peaks.

The FTIR spectrum of voriconazole (Figure 2A) shows typical characteristic peaks at 3,199.76 cm^−1^ (indicative of O-H stretching), 1,497.90 to 1,589.85 cm^−1^ (C=C stretching), 1,352.59 to 1,245.11 cm^−1^ (aryl C-N stretching), and at 1,409.00 to 1,132.69 cm^−1^ (C-F stretching) (Khare et al., 2016; Sun et al., 2016). The spectrum of physical mixture of voriconazole, DPPC, HSPC and CHL (Figure 2B) depicts no major shifting of functional peaks due to the drug. The spectrum of the optimized LNVs (Figure 2C) portrays the characteristic peaks of HSPC, i.e., C-H stretching vibration of long fatty acid chain at 2,917.89 to 2,852.36 cm^−1^ (Zhao et al., 2019) and of DPPC at 3,499.78 to 3,346.36 cm^−1^ (O-H stretching) and 1,242.40 to 1,099.29 cm^−1^ (PO2- groups) (Pawlikowska-Pawlęga et al., 2013). The characteristic peak due to the drug in LNVs is diminished compared to pure drug peaks, indicating the dispersion of drug in the phospholipid matrix.

#### P-XRD Studies

Voriconazole (Figure 2D) exhibits distinctive peaks at 12.6°, 13.8°, 15.9°, 16.5°, 17.4°, 19.8°, 26.1° and 28.1° 2θ. The physical mixture in Figure 2E manifests distinct, though less sharp, peaks at 6.2°, 8.7°, 10.5°, 13.8°, 21.2°, 21.8° and at 25.1° 2θ, while the diffractogram of LNVs in Figure 2F depicts a small and broad peak at 21.2° 2θ only.

#### *In Vitro* Drug Release Studies

**FIGURE 3:**
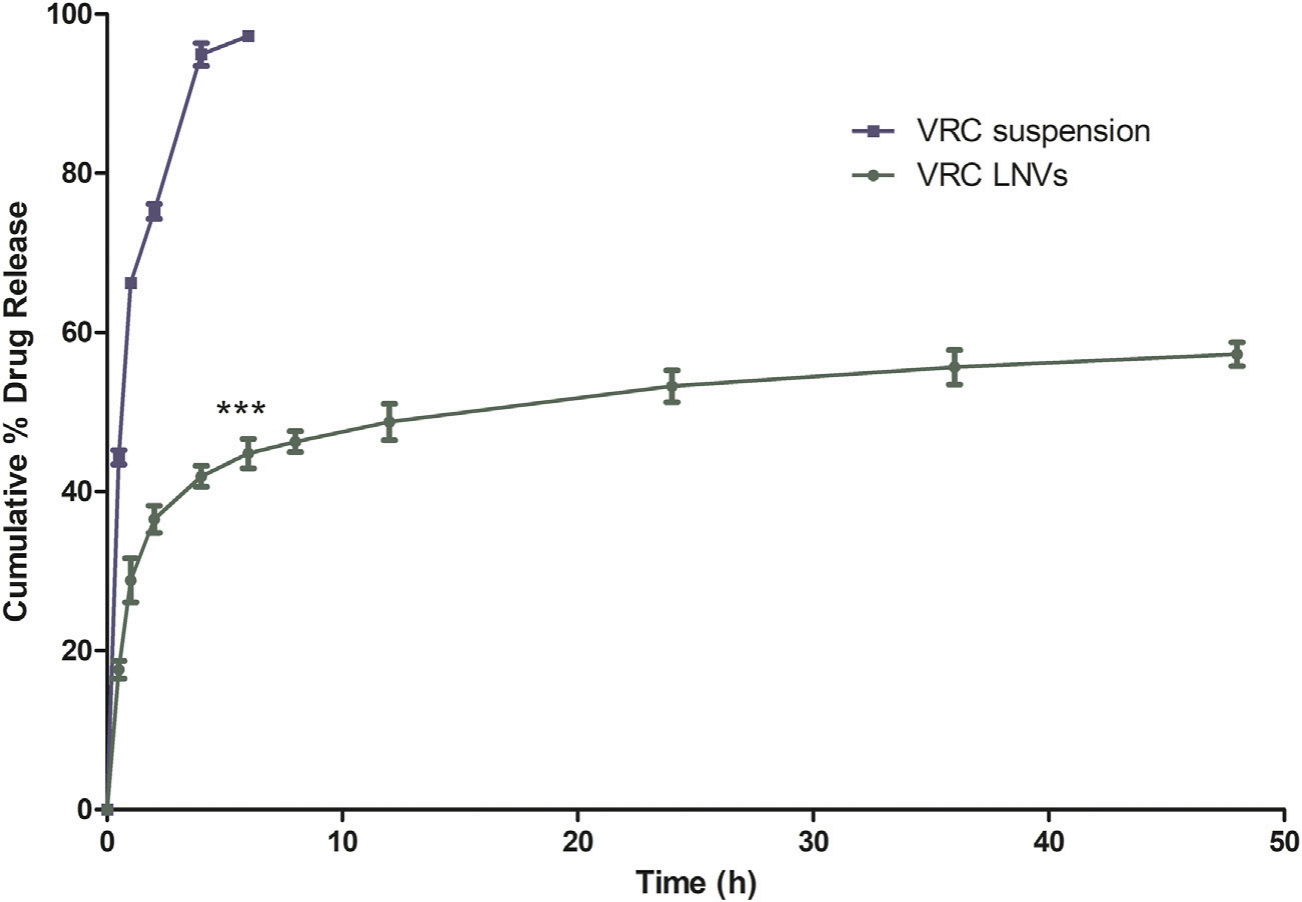
*In vitro* cumulative drug release profile of voriconazole (VRC) suspension and optimized lipid nanovesicles (LNVs) in 20 ml phosphate buffer saline pH 7.4 for a time period of 48 h. Error bars represents Mean ± SD values (*n* = 3). The asterisks represent statistically significant difference (***p < 0.0001) between the drug release profile of VRC from suspension and of LNVs at the same time period, i.e., of 6 h.

Voriconazole exhibited 97.27 ± 1.20% drug release in 6 h, whereas the corresponding LNVs showed an initial burst release (i.e., 36.52 ± 2.95%) for first 2 h, followed by extended release profile (*i*.e., 57.24 ± 2.61%) till 48 h (Figure 3 **)**. The drug release kinetic model fitting depicted Korsemeyer-Peppas to be the best-fitted model with a high coefficient of correlation (R = 0.932, *p* < 0.05; Supplementary Table S3)**.** The computed release exponent (*n* = 0.222), being magnitudinally less than the threshold limit of 0.45, construed Fickian diffusion as the underlying drug release mechanism.

#### Microdroplet Size Analysis Using Laser Diffraction

**FIGURE 4:**
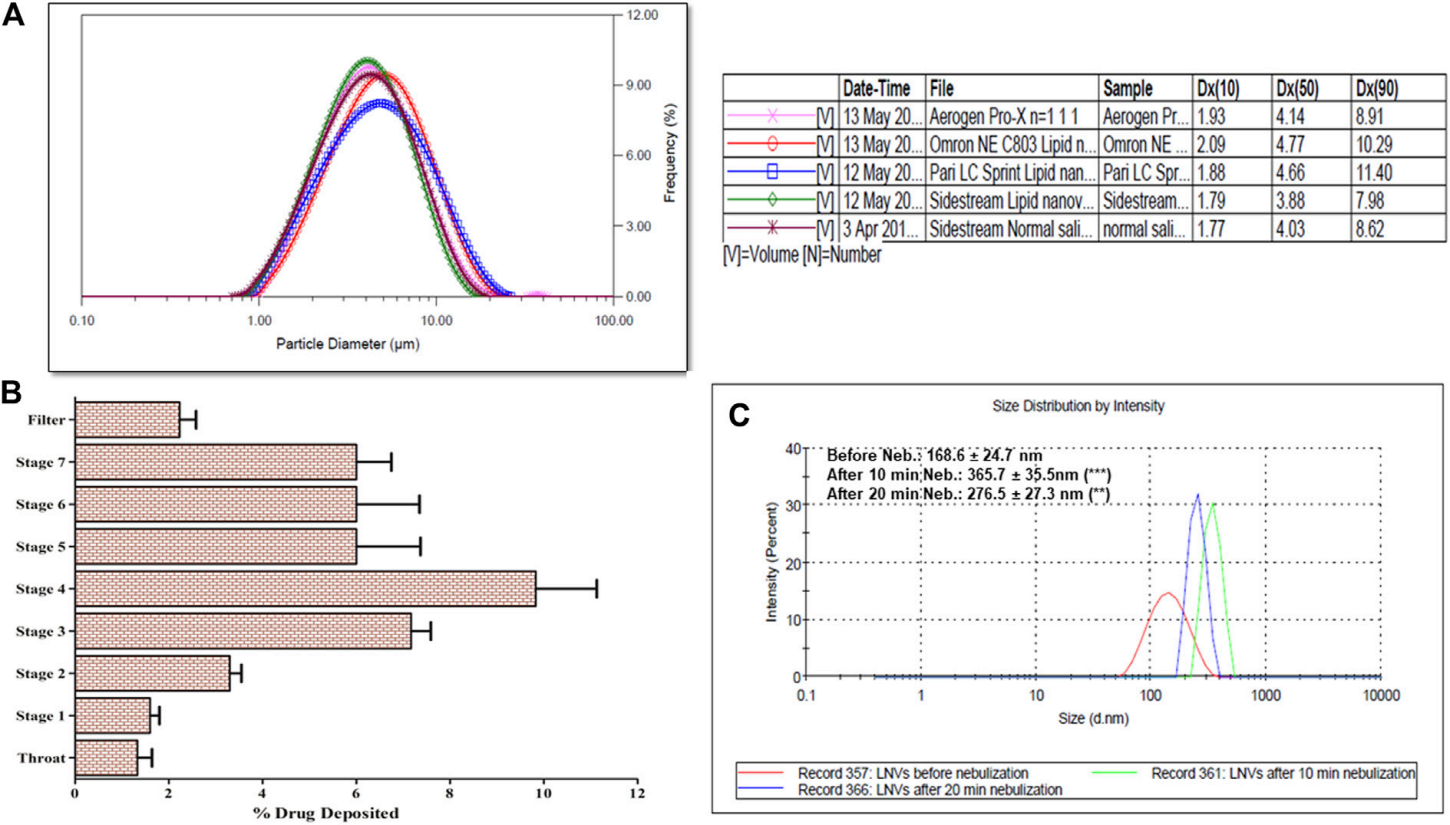
**(A)** Microdroplet aerosol investigation of lipid nanovesicles (LNVs) employing Spraytec laser diffraction using jet and mesh nebulizers, with a lens of 300 mm focal length. Lowest Dx (50) value was observed with Sidestream nebulizer, which is significantly (*p* < 0.05) different from Omron and Pari nebulizers, while “ns” indicates insignificant difference (*p* > 0.05) from Aerogen solo; **(B)** Next-generation impactor studies of LNVs employing Sidestream nebulizer at a flow rate of 15 L min^−1^, indicating maximum deposition on Stage 3 and Stage 4 of impactor; **(C)** Stability profile of LNVs, before and after nebulization of 20 min; Data represent mean ± SD values (*n* = 3). Data sets were compared between results, before and after nebulization (**p < 0.005, ***p < 0.001).

The microdroplet size-distribution analysis of different nebulizers demonstrated the lowest Dx (50) value with Sidestream nebulizer, which is significantly different (*p* < 0.05) from the Dx (50) values of Omron and Pari nebulizers, but quite similar to those of Aerogen solo nebulizer (Table 2, Figure 4A). Significant variation (*p* < 0.05) was also observed in FPF value of Sidestream nebulizer, i.e., droplet size ≤5 µm (65.98 ± 0.45%) and 3 µm (33.06 ± 0.38%), when compared with other nebulizers. The corresponding values of VMD and GSD were observed as 3.90 ± 0.13 and 1.72 ± 0.12 µm, respectively. No significant difference (*p* > 0.05) was noticed in microdroplet sizes generated using Sidestream nebulizer between LNVs dispersion and normal saline solution. Accordingly, the results obtained using Sidestream nebulizer were selected for further experiments.

**TABLE 2:** A comparative account on performance characteristics of different nebulizers. Data expressed as mean ± SD (*n* = 3).

Parameters	Aerogen solo	Omron NE-C803	PARI LC	Side stream
Dv 10 (µm)	1.99 ± 0.23	2.09 ± 0.14	1.86 ± 0.10	1.80 ± 0.12
Dv 50 (µm)	4.23 ± 0.17	4.83 ± 0.16	4.60 ± 0.16	3.90 ± 0.13
Dv 90 (µm)	8.94 ± 0.23	10.46 ± 0.14	11.21 ± 0.32	8.04 ± 0.17
Span	1.65 ± 0.13	1.73 ± 0.13	2.02 ± 0.12	1.60 ± 0.14
<5 µm	60.85 ± 1.14	52.13 ± 0.80	54.47 ± 0.77	65.98 ± 0.45
1–3 µm	28.78 ± 0.65	23.61 ± 0.71	27.81 ± 0.41	33.06 ± 0.38
GSD	1.72 ± 0.18	1.79 ± 0.14	2.05 ± 0.19	1.72 ± 0.12

*Dv (10), microdroplets below which 10% spray lies; Dv (50), microdroplets below which 50% spray lies; Dv (90), microdroplets below which 90% spray lies; GSD, geometric standard deviation.

#### *In vitro* Aerosol Performance Using NGI

Figure 4B illustrates the pulmonary deposition pattern of LNVs during different stages of NGI. Maximal deposition was observed on Stage 4 (3.3 μm cut-off) and Stage 3 (5.4 μm cut-off) of the NGI, with MMAD and GSD as 3.25 ± 0.41 µm and 2.50 ± 0.58, respectively. A total of 68.66 ± 7.64% dose was discharged from nebulizer with FPF as 49.00 ± 3.46% (*i.e.,* droplet size <5 µm) and 39.99 ± 2.21% (*i.e.,* droplet size <3 µm).

#### Stability of LNVs Before and After Nebulization

Globule size of LNVs in the nebuliser reservoir after air-jet nebulization indicated 2.7-folds (365.7 ± 35.5 nm; *p* < 0.001) augmentation in particle size after 10 min, while only 2.1-folds (276.5 ± 27.3 nm; *p* < 0.005) increase was observed after 20 min of nebulization (Figure 4C **).**

#### Surface Activity Experiments

**FIGURE 5:**
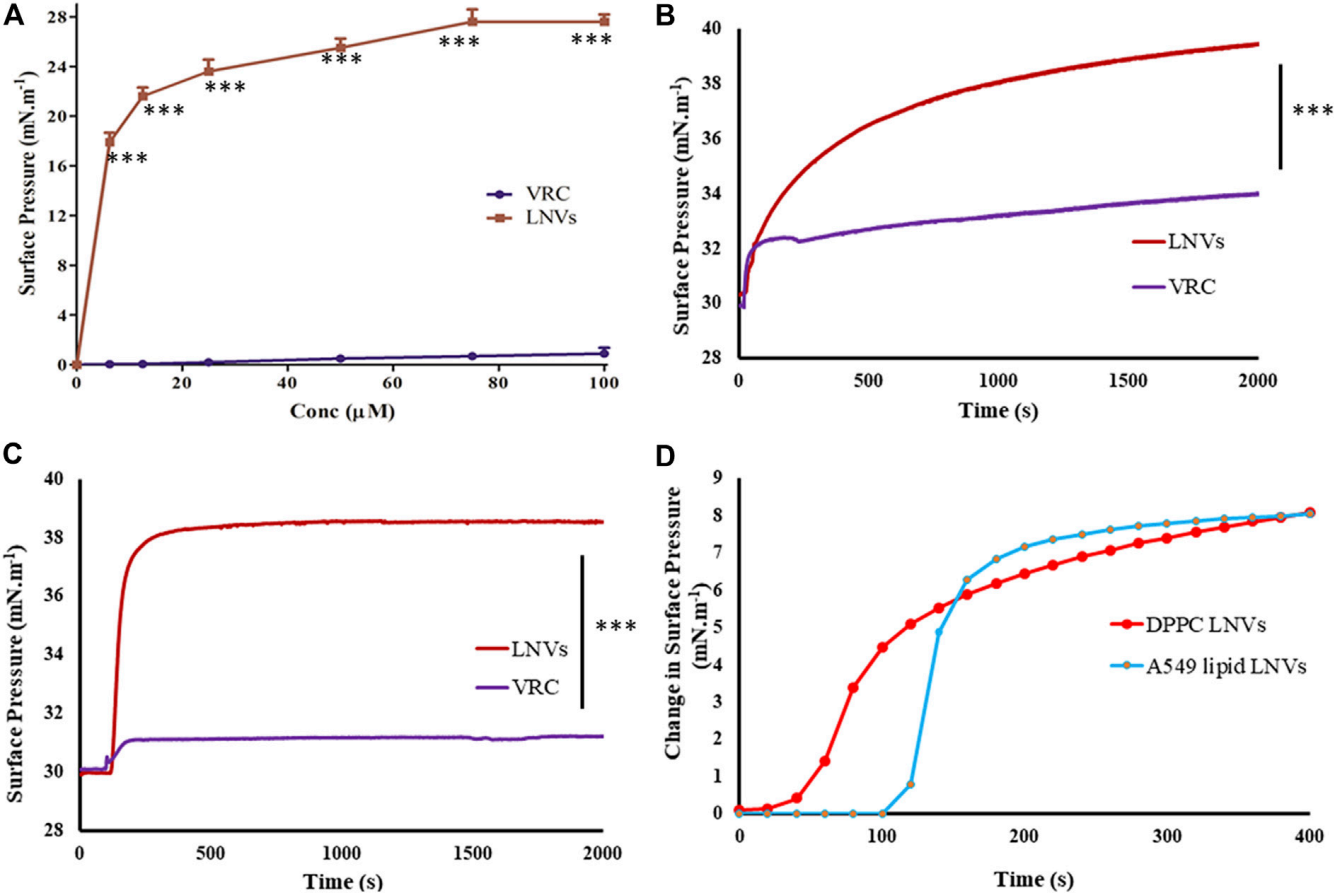
**(A)** Surface activity profile of voriconazole (VRC, purple) and its lipid nanovesicles (LNVs, red) at air/water subphase employing Langmuir Blodgett trough filled with PBS pH 7.4 (10 mM) and adjusted to 15 cm^2^ area; lipid monolayer interaction statistics of VRC and LNVs at **(B)** DPPC and at **(C)** A549 cells lipid extract (0.5 mM) at 30 mN m^−1^ of pressure; **(D)** Initial change in the surface pressure observed with LNVs intercalation into DPPC and A549 cell lipid monolayers for time-duration of 400 s. Data represent mean ± SD values (*n* = 3). Data sets were compared with pure voriconazole (***p < 0.0001).

Figure 5A portrays increasing surface pressure with corresponding drug concentrations, until it reaches saturation. Minimum voriconazole concentration of 75 µM was required to saturate the air/water interface for LNVs.

##### Interaction of Voriconazole and Lipid Nanovesicles With Lipid Monolayers

Interaction of LNVs with DPPC or lipid extract monolayers followed hyperbolic kinetics, induced maximal surface pressure increase of 9.39 ± 0.47 mN.m^−1^ with DPPC monolayer and 8.41 ± 0.48 mN m^−1^ with A549 cells lipid monolayer (Figures 5B,C). On the other hand, minimal values (<3 mN m^−1^) of surface pressure differential was observed with voriconazole. Figure 5D presents the initial time-points (upto 400s) of Figures 5B,C showing faster intercalation of LNVs into DPPC monolayer as compared to A549 lipid monolayer.

### Cell Culture Studies

#### Safety Studies

**FIGURE 6:**
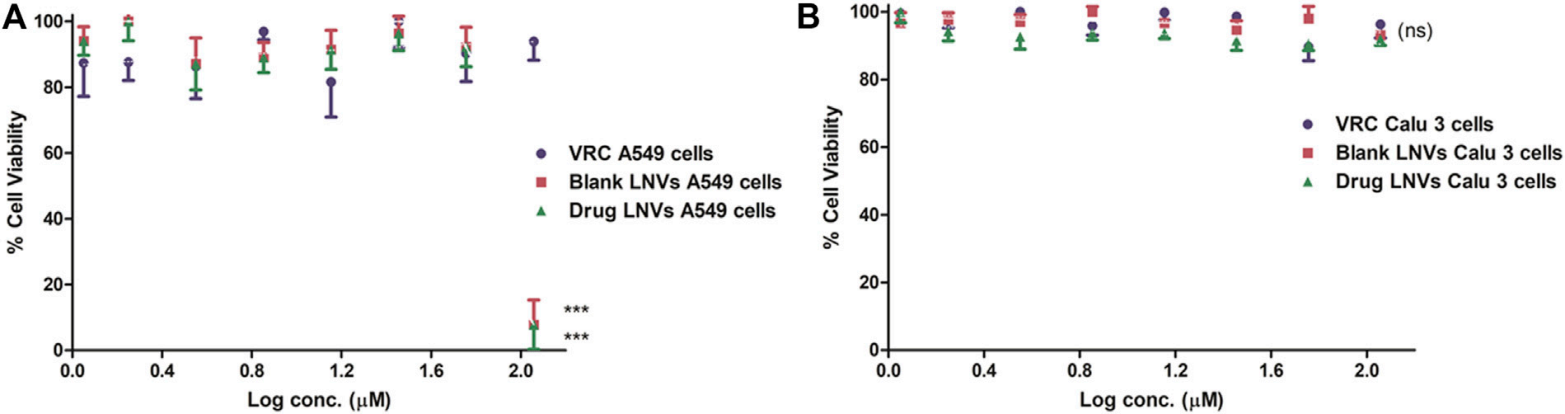
Cell viability assay employing Presto Blue on lung epithelial cell lines **(A)** A549 and **(B)** Calu 3 cell lines for a time-duration of 48 h. Data represent mean ± SD values (*n* = 3). The asterisks represent statistically significant difference (***p < 0.0001) between the cell viability of drug compared to blank and drug-loaded formulation in A549 cells at log conc. of 2 μM, while “ns” indicates an insignificant (*p* > 0.05) difference between the tested groups in Calu 3 cells (ns).

Over 90% cell viability was exhibited by LNVs w.r.t. Calu 3 cells, after treating with the highest tested concentration (i.e., 114 µM). In case of A549 cells, however, > 90% viability was observed at 57 μM, which got reduced to <20% at 114 μM, as illustrated in Figure 6.

#### Qualitative and Quantitative Uptake Studies

**FIGURE 7:**
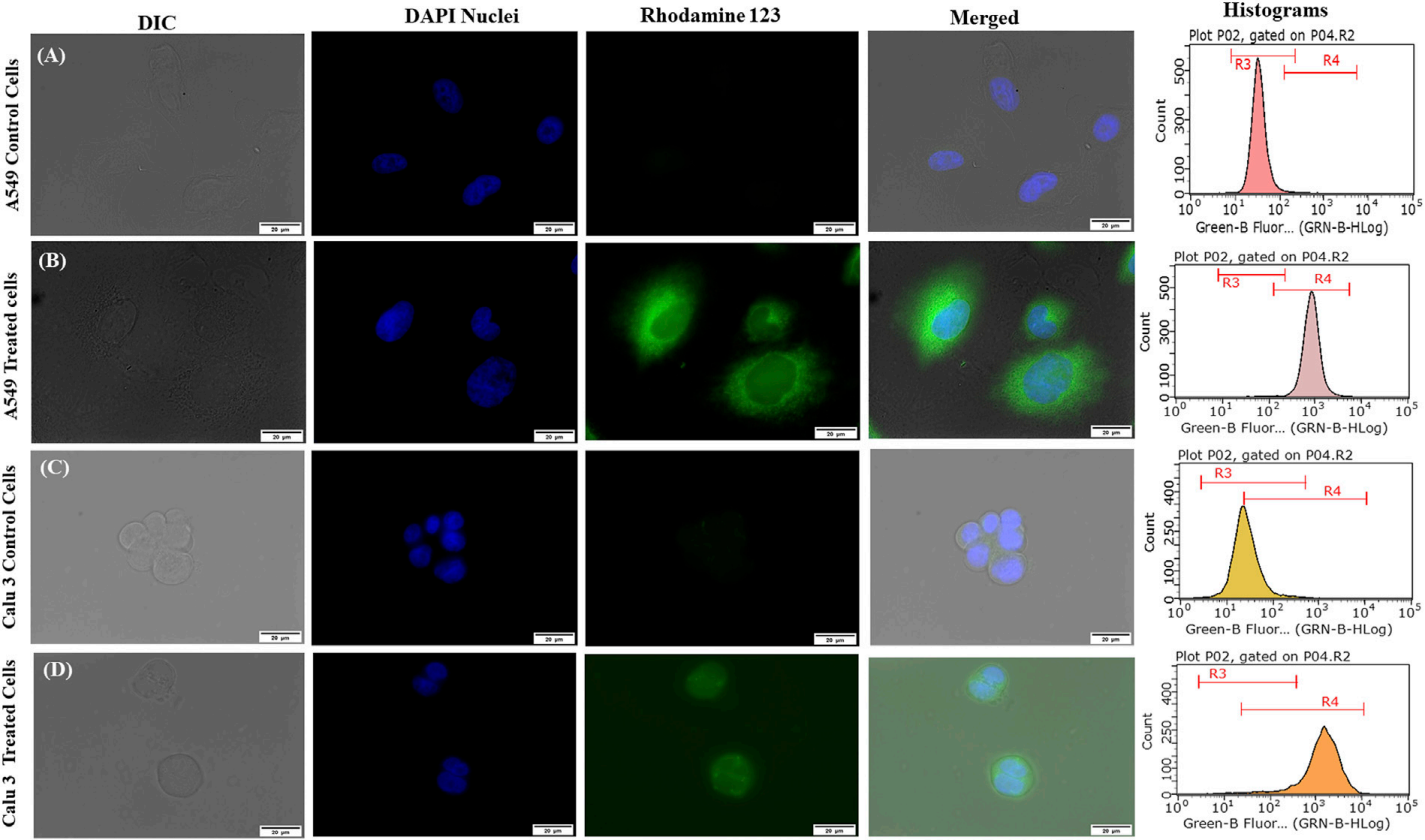
Fluorescent microscopic images and histograms of Rhodamine 123-loaded lipid nanovesicles (LNVs) with green (Rhodamine 123) and blue (DAPI) fluorescence in **(A)** A549 control cells **(B)** A549-treated cells, **(C)** Calu 3 control cells, **(D)** Calu 3-treated cells.

**FIGURE 8:**
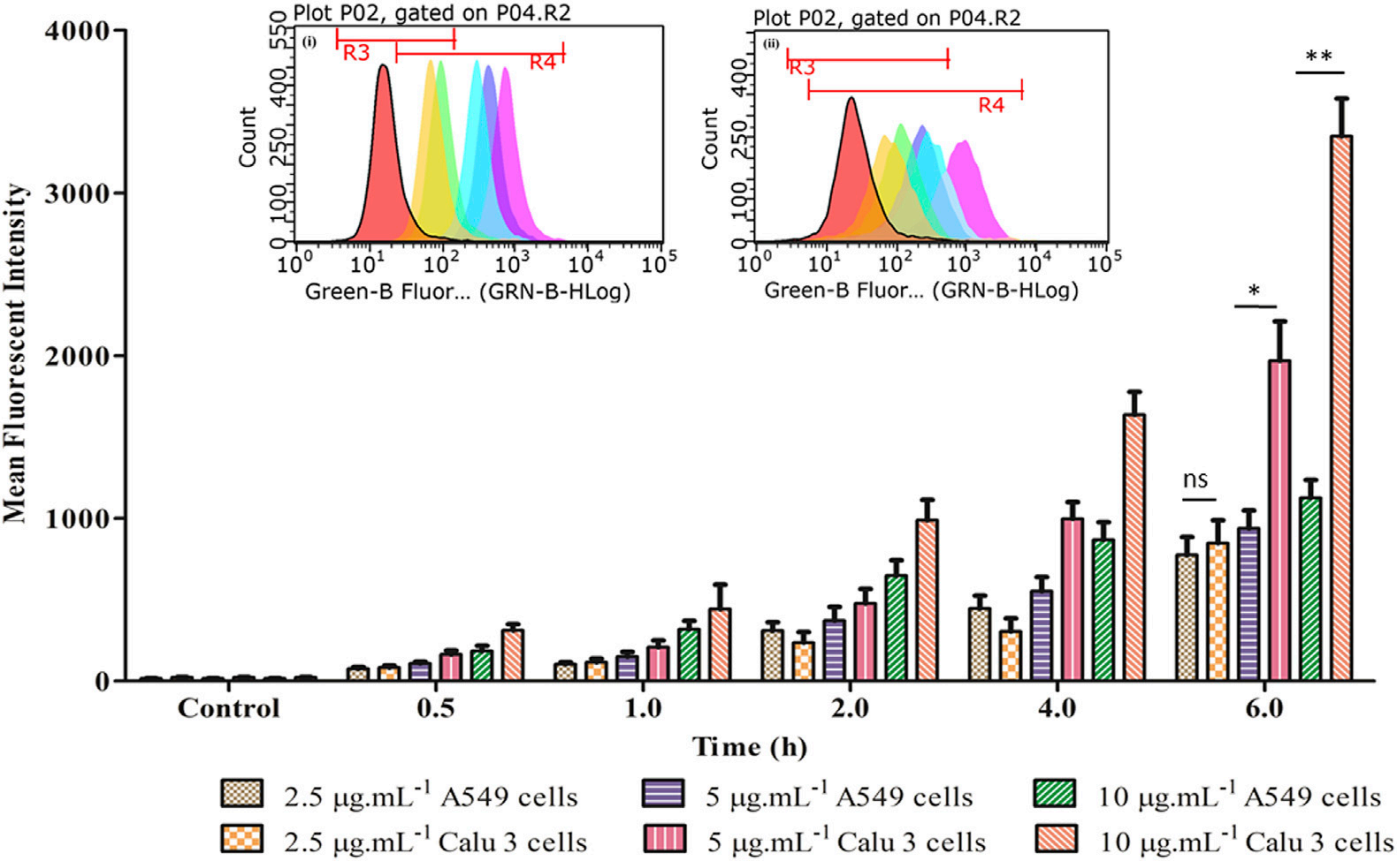
Time- and concentration-dependent profile of fluorescent intensity of LNVs in A549 and Calu 3 cells. The corresponding inset displays the flow cytometry data of LNVs (2.5 μg ml^−1^) for a time duration of 6 h in (i) A549 and (ii) Calu 3 cell lines, respectively. Data represent mean ± SD values (*n* = 3). The asterisks represent statistically significant difference (*p < 0.01, **p < 0.0001), while “ns” indicates an insignificant (*p* > 0.05) difference between the groups tested.

Figures 7A–D portrays considerable internalization of LNVs into airway cell lines w.r.t. control cells. Substantial green fluorescence within A549 as well as in Calu 3 cells indicated co-localization of Rh-123 LNVs after 4 h of incubation period. Internalization of LNVs in the airway epithelial cells, as shown in Figure 8, increases linearly towards right in a time- and concentration-dependent manner, using both of the lung epithelial cells.

#### Mechanistic Pathway Studies

**FIGURE 9:**
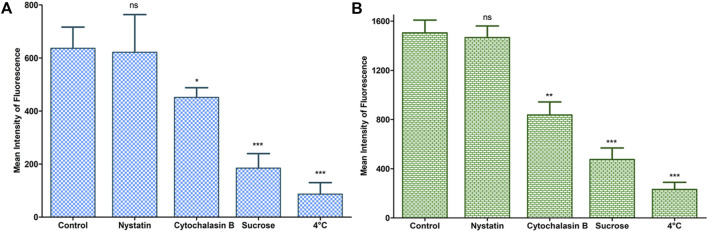
Study of endocytic mechanistic pathways during cellular uptake of LNVs in A549 **(A)** and Calu 3 **(B)** cell lines using a flow cytometer. Data represent mean ± SD values (*n* = 3). The asterisks represent statistically significant difference (***p < 0.0001, **p < 0.005, *p < 0.05), while “ns” indicates an insignificant (*p* > 0.05) difference between the control A549 and the tested Calu 3 cells groups.

The bar charts in Figures 9A,B depict the endocytic mechanistic pathways underlying the internalization of LNVs in A549 and Calu 3 cells. Clathrin (or sucrose inhibition) and macropinocytosis (or cytochalasin B inhibition) pathways were found to be the prime mechanisms associated with the internalization of LNVs in airway epithelial cells. Nearly 3.5- and 3.2-folds reduction (*p* < 0.0001) in LNVs uptake with clathrin-mediated pathway, and 1.4- and 1.8-folds reduction *p* < 0.05; *p* < 0.005) with macropinocytosis pathway, was observed using A549 and Calu 3 cells, respectively, in the presence of pharmacological inhibitors for these pathways. Inconsequential effect (*p* > 0.05) on internalization, however, was noticed with the caveolae/lipid rafts pathway (*i.e.,* nystatin) on both the cell lines. Effect of temperature on the uptake of Rh-LNVs unearthed the involvement of energy-dependent endocytic pathway for the entrance of LNVs into the airway epithelial cells, as a highly significant reduction of 6 to 7-folds (*p* < 0.0001) was noticeable vis-à-vis the control cells.

### Antifungal Efficacy

MIC values for LNVs (Table 3 **)** were found to be nearly 2-folds lower for *Aspergillus fumigatus* and *Candida parapsilosis,* while quite similar to those of *Aspergillus flavus* and *Candida krusei,* when compared to pure drug.

**TABLE 3:** Values of minimum inhibitory concentration (MIC) of voriconazole (VRC) and its lipid nanovesicles (LNVs) against different fungal isolates (*n* = 3).

Sr No	Fungal strain	Lab ID	MIC values (µg.ml^−1^)		
			VRC	Blank LNVs	Drug LNVs
1	*Candida krusei*	CK-6258	0.23	14.84	0.23
2	*Candida parapsilosis*	CP-22019	0.05	14.84	0.03
3	*Aspergillus flavus*	ATCC-204304	0.92	14.84	0.92
4	*Aspergillus fumigatus*	ATCC-204305	0.92	14.84	0.46

### Plasma and Lung Pharmacokinetic Studies

Supplementary Figure S5A depicts the lung pharmacokinetic profiles of voriconazole and LNVs after their nebulization for 20 min in Balb/c mice, while the corresponding inset displays percent change in the pharmacokinetic parameters between the two treatment groups. LNVs showed significantly improved lung retention potential of voriconazole upto 24 h, over the commercial drug formulation, wherein the drug wasn’t even detectable in lungs after 12 h. As much as 4.0-folds (*p* < 0.005), 4.2-folds (*p* < 0.005), 3.3-folds (*p* < 0.05), 2.4-folds (*p* < 0.05) and 1.5-folds (*p* > 0.05) enhancement was observed in the values of AUC_0-24_, AUC_0-**∞**,_ T_max_, MRT and C_max_, respectively (Table 4).

**TABLE 4:** Pharmacokinetic parameters (Mean ± SD, *n* = 3) of voriconazole (VRC) and lipid nanovesicles following 20 min of nebulization.

Pharmacokinetic parameter	Lung pharmacokinetics		Plasma pharmacokinetics	
	VRC solution	VRC LNVs	VRC solution	VRC LNVs
C_max_ (μg.ml^−1^)	13.43 ± 3.10	18.69 ± 3.35	6.81 ± 1.11	9.15 ± 2.45
T_max_ (h)	0.17 ± 0.00	0.57 ± 0.40	0.39 ± 0.19	1.33 ± 0.48
AUC_0-24_ (μg.mL^−1^.h)	36.38 ± 5.40	143.81 ± 32.26	33.89 ± 5.05	91.21 ± 14.25
AUC_0-∞_ (μg.ml^−1^.h)	41.77 ± 6.75	173.72 ± 38.83	35.94 ± 6.11	113.66 ± 26.80
MRT (h)	5.27 ± 1.31	12.64 ± 3.42	7.13 ± 1.03	13.95 ± 3.49

Plasma level profile of voriconazole (Supplementary Figure S5B **)**, however, demonstrated relatively modest improvement in AUC_0-24_, AUC_0-**∞**_ **,** T_max_, MRT and C_max_ values over marketed formulation, accounting for augmentation of almost 2.7-folds (*p* < 0.005), 3.2-folds (*p* < 0.005), 3.4-folds (*p* < 0.05), 2.0-folds (*p* < 0.05) and 1.3-folds (*p* > 0.05), respectively.

## Discussion

In the current studies, inhalable LNVs of voriconazole were prepared as an alternative to the conventional therapeutic regimens employing lung-endogenous phospholipids. DPPC, being biomimetic to lung surfactant, was chosen to reduce air-water interfacial tension and to prevent lung collapse. HSPC, a longer aliphatic chain phosphatidylcholine, was added to improve membrane stability and prevent any drug leakage at body temperature (Chen et al., 2013). Cholesterol was added to impart fluidity and stability to the membrane of developed nanostructured system (Rudokas et al., 2016; Tagami et al., 2017), as nebulization is usually associated with generation of strong shear forces that could alter the intrinsic properties of the studied system. In the current studies, the chosen phospholipids exhibit the ability to integrate into single phase in the vesicular membrane through electrostatic interactions, as the cationic portion of HSPC tends to attract the anionic portion of DPPC (Chen et al., 2013).

Nanovesicular characteristics of the prepared LNVs were confirmed through the particle size analysis and FESEM imaging. The polygonal shape observed during the FESEM imaging can be attributed to the usage of phospholipids (DPPC and HSPC) with fully-saturated fatty-acids, while their smooth appearance could be rationally ascribed to the influence of cholesterol (Mahmud et al., 2016). The FTIR studies indicate either no or weak interactions, thereby ruling out any incompatibility between the drug and the selected excipients. P-XRD is documented to be a non-destructive technique to provide definitive evidence about the crystalline characteristics of the samples (Rahman et al., 2010). A number of sharp peaks are apparent in the diffractogram of pure drug (i.e., Figure 2D), indicating its crystalline nature, which is in concurrence with various literature reports (Khare et al., 2016; Chen et al., 2020). However, the physical mixture (Figure 2E) resulted in a relatively less intense crystalline peaks of drug due to the superposition of excipient peaks. The diffractograms of raw DPPC and HSPC (Supplementary Figure S6) show a strong characteristic peak at 21.2° 2θ, which is quite apparent in the diffractogram of physical mixture. While comparing with the diffractograms of pure drug and physical mixture, the diffractogram of LNVs (Figure 2F) shows a quite small and broad peak at an angle 21.2° 2θ. This can be ascribed to the amorphous nature of the nano-sized material (Fawcett et al., 2015). As the amorphous samples do not produce diffraction patterns owing to lack of periodic array with long-range order, their XRD pattern generally features broad and poorly defined amorphous humps (Speakman, 2011). Thus, the nanostructured lipidic vesicles appear to have altered the physical state of the drug molecule from the erstwhile crystalline nature to an amorphous state. Drug release studies showed a biphasic pattern with burst release phenomenon in initial 2 h, followed by relatively regulated release profile till 48 h. While the initial burst could be attributed to superficially adsorbed drug onto LNVs, subsequent controlled release can be assigned to deep encapsulation of drug molecule in the vesiclar structure and formation of gel-like structure at 37°C by high-transition temperature lipids (Wauthoz and Amighi, 2014; Elhissi, 2017).

For attaining ideal lung delivery, LNVs were further converted into microdroplets employing the best suited nebulizer, i.e., Sidestream. Greater lung deposition could be achieved with microdroplets exhibiting MMAD and VMD, ranging between 1 and 5 μm, construing criticality of particle size in affecting lung deposition (Labiris and Dolovich, 2003). Analysis of NGI and Spraytec data revealed MMAD (3.25 ± 0.41 µm) and VMD (3.90 ± 0.13 µm) values of <5 μm, construing their definitive ability to overcome the first physical barrier of airways and drug targeting, majorly *via* the sedimentation and diffusion mechanisms (Labiris and Dolovich, 2003; Kaur et al., 2019). Further, strong shear forces of the nebulizer during the process of nebulization resulted in higher particle size after 10 min of nebulization as compared to 20 min, which could be ascribable to the fusion, aggregation or reformation of the vesicles in the main reservoir (Nimmano et al., 2018).

When delivered to lungs, microdroplets come in contact with biochemical or alveolar barrier, *i.e.,* pulmonary surfactant monolayer, that maintains surface tension at the air-water interface and aids in breathing (Murgia et al., 2014). Herein, *in vitro* interaction(s) of LNVs with A549 lipid and DPPC monolayers, mimicking pulmonary surfactant monolayer, indicated their superior surface-active potential vis-à-vis the pure drug. Moreover, their stability with the monolayer phospholipids could be hypothesized to their structural similarity with the membrane, thus facilitating their intercalation and stabilization into the membrane (Joshi et al., 2014; Li et al., 2015). The fast intercalation, observed in Figure 5D into DPPC monolayer as compared to A549 cell monolayer, could be assigned to the diverse origin and nature of phospholipids.

After crossing pulmonary surfactant monolayer, it is the safety and uptake potential, along with plausible mechanistic pathways through the cellular barrier, which plays imperative role in inhalational drug delivery. Accordingly, the prepared LNVs were observed to exhibit promising cytocompatibility with airway epithelial cell lines. However, the formulation was found to be safer on Calu 3 cells (114 µM) than on A549 cells (57 µM), attributable to the varied morphological features of the cell lines. Cellular uptake studies revealed augmentation in internalization of LNVs at higher concentrations and for prolonged time-periods. As nanostructured systems can internalise into the cells by different pathways, successful investigations on the underlying mechanistics signified primarily clathrin- and modestly macropinocytosis-pathways to be responsible for the entry of LNVs inside the airway cells. The positive surface charge of LNVs could have resulted in electrostatic interactions and promoted its internalization by clathrin-pathways, though macropinocytosis has the ability to internalize both positive and negative vesicles (Behzadi et al., 2017; Khurana et al., 2018).

Further, *in vitro* efficacy of LNVs on the diverse laboratory fungal strains revealed either decreased or unchanged MIC values. This could possibly be ascribed to either nanostructured characteristics of the formulation or morphological features of the tested fungal strains.

*In vivo* lung pharmacokinetic studies on LNVs in Balb/c indicate their significantly improved drug retention potential compared to marketed formulation upon nebulization. This could be designated to notable augmentation in the pharmacokinetic parameters like AUC and MRT. LNVs-embedded microdroplets, being smaller than 5 µm, get easily deposited in the respiratory airways. Markedly superior surface-active potential of LNVs over pure drug potentially facilitates its interaction(s) with pulmonary surfactant monolayer and promotes its intercalation into the alveolar epithelial cells (Garcia-Mouton et al., 2019), as demonstrated by Langmuir Blodgett studies. Lower surface activity has been associated with faster removal process owing to continuous compression and expansion of surfactant monolayer through breathing cycles (Garcia-Mouton et al., 2019), which might be responsible for the fast removal of voriconazole from lungs. Further, LNVs being well-below 500 nm, possess high potential to minimize their macrophageal clearance and to enhance their deposition in the respiratory airways through their diffusive mobility (Patlolla et al., 2010).

Peak plasma levels of voriconazole were observed after 1.3 h of LNVs nebulization compared to 0.39 h of marketed formulation, apparently depicting markedly better retention and slower diffusion of the former from the lungs. Entry of voriconazole into plasma is anticipated to depend upon its release from LNVs and its diffusion across membrane. Presence of thin epithelial membrane and large alveolar surface area in the respiratory zone tends to enhance drug diffusion into systemic circulation, thereby accounting for escalation in plasma AUC values. Albeit some tracheobronchial deposition of voriconazole is expected following the nebulization of LNVs, systemic drug absorption is likely to be minimal owing to thicker epithelial membrane and lower surface area (Patlolla et al., 2010).

## Conclusion

The current work successfully demonstrates the distinct potential of LNVs to target the respiratory airways. Usage of lung endogenous phospholipid(s), eventually resulting in significantly improved membrane stability during nebulization, modulated drug release profile and improved aerodynamic characteristics. Furthermore, safety and biocompatibility with pulmonary surfactant monolayer and airway cell lines, along with improved drug retention potential in mice lungs, corroborate the immense potential of inhalable LNVs. The favourable outcomes, and the knowledge gained thereof from the present research work, could form definitive basis of translational research for the treatment and prophylaxis of various fungal lung infections. Besides, the leads drawn from the present inhalational drug delivery work could also be successfully extrapolated for effective therapeutic management of various other pulmonary infections.

## Data Availability

The original contributions presented in the study are included in the article/Supplementary Material, further inquiries can be directed to the corresponding author.

## Abbreviations

A549, adenocarcinoma human alveolar epithelial cells; ARDS, acute respiratory distress syndrome; BBD, Box-Behnken design; Calu 3, human bronchial epithelial cells; CAPA, covid-19 associated pulmonary aspergillosis; CMAs, critical material attributes; CPPs, critical process parameters; CQAs, critical quality attributes; DPPC, dipalmitoylphosphatidylcholine; DR_8h_, cumulative drug release at 8 h; ED, emitted dose; EE, entrapment efficiency; FFD, fractional factorial design; FPF, fine particle fraction; GSD, geometric standard deviation; HSPC, hydrogenated soya-phosphatidylcholine; IPA, invasive pulmonary aspergillosis; LNVs, lipid nanovesicles; MMAD, mass median aerodynamic diameter; NGI, next-generation impactor; PDI, polydispersity index; PS, particle size; QbD, quality-by-design; Rh-123, rhodamine 123; VMD, volume mean diameter; ZP, zeta potential.
