# Supplementary material for: Aerosolizable Lipid-Nanovesicles Encapsulating Voriconazole Effectively Permeate Pulmonary Barriers and Target Lung Cells
Source: Front Pharmacol. 2022 Mar 10;12:734913. doi: 10.3389/fphar.2021.734913 (PMC8982086; doi:10.3389/fphar.2021.734913)
Supplement: Supplementary file 1 [file DataSheet1.docx]

| **Run** | **DPPC**  **(mg)** | **HSPC**  **(mg)** | **CHL**  **(mg)** | **Temperature**  **(°C)** | **Sonication time**  **(sec)** |
| --- | --- | --- | --- | --- | --- |
| 1. | -1 | 1 | 1 | -1 | -1 |
| 2. | 1 | -1 | 1 | -1 | 1 |
| 3. | 1 | 1 | 1 | 1 | 1 |
| 4. | -1 | -1 | -1 | 1 | 1 |
| 5. | 1 | -1 | -1 | -1 | -1 |
| 6. | 1 | 1 | -1 | 1 | -1 |
| 7. | -1 | 1 | -1 | -1 | 1 |
| 8. | -1 | -1 | 1 | 1 | -1 |
| **Factors** | | **Low (-1)** | | **High (+1)** | |
| DPPC (mg) | | 40 | | 120 | |
| HSPC (mg) | | 40 | | 120 | |
| CHL (mg) | | 10 | | 30 | |
| Temperature (°C) | | 55 | | 60 | |
| Sonication time (sec) | | 60 | | 120 | |

**Table S1.** Design matrix for factor screening of lipid nanovesicles of voriconazole as per Fraction Factorial Design

*DPPC: Dipalmitoylphosphatidylcholine; HSPC: Hydrogenated soya-[phosphatidylcholine](https://www.sciencedirect.com/topics/pharmacology-toxicology-and-pharmaceutical-science/egg-lecithin); CHL: Cholesterol

**Table S2.** Design matrix for systematic optimization of lipid nanovesicles of voriconazole as per Box-Behnken Design

| **Formulation code** | **CRITICAL MATERIAL ATTRIBUTES (CMAs)** | | |
| --- | --- | --- | --- |
|  | **HSPC** | **DPPC** | **CHL** |
| 1 | -1 | 1 | 0 |
| 2 | 0 | 0 | 0 |
| 3 | 0 | 0 | 0 |
| 4 | 0 | 0 | 0 |
| 5 | 0 | 1 | 1 |
| 6 | 1 | 0 | 1 |
| 7 | -1 | 0 | 1 |
| 8 | 1 | 1 | 0 |
| 9 | 0 | -1 | -1 |
| 10 | 1 | 0 | -1 |
| 11 | 0 | -1 | 1 |
| 12 | -1 | -1 | 0 |
| 13 | -1 | 0 | -1 |
| 14 | 1 | -1 | 0 |
| 15 | 0 | 0 | 0 |
| 16 | 0 | 0 | 0 |
| 17 | 0 | 1 | -1 |
| **Factors** | | **Low (-1)** | **High (+1)** |
|  |  |  |  |
| HSPC (mg) | | 40 | 120 |
| DPPC (mg) | | 40 | 120 |
| CHL (mg) | | 10 | 30 |

*DPPC: Dipalmitoylphosphatidylcholine; HSPC: Hydrogenated soya-[phosphatidylcholine](https://www.sciencedirect.com/topics/pharmacology-toxicology-and-pharmaceutical-science/egg-lecithin); CHL: Cholesterol

**Table S3:** Drug release kinetic modeling for the optimized lipid nanovesicles of voriconazole

| Model | Model coefficient | R |
| --- | --- | --- |
| Zero-order | 0.556 | 0.768 |
| First-order | -0.004 | 0.818 |
| Higuchi | 4.911 | 0.880 |
| Korsemeyer-Peppas | 0.226 | 0.932 |

1. B.

C. D.

E. F.

G. H.

**Figure S1:** Half-normal plots and Pareto charts depicting the influence of MAs and PPs on the CQAs of lipid nanovesicles of voriconazole, (A-B) particle size; (C-D) PDI; (E-F) zeta potential and (G-H) entrapment efficiency


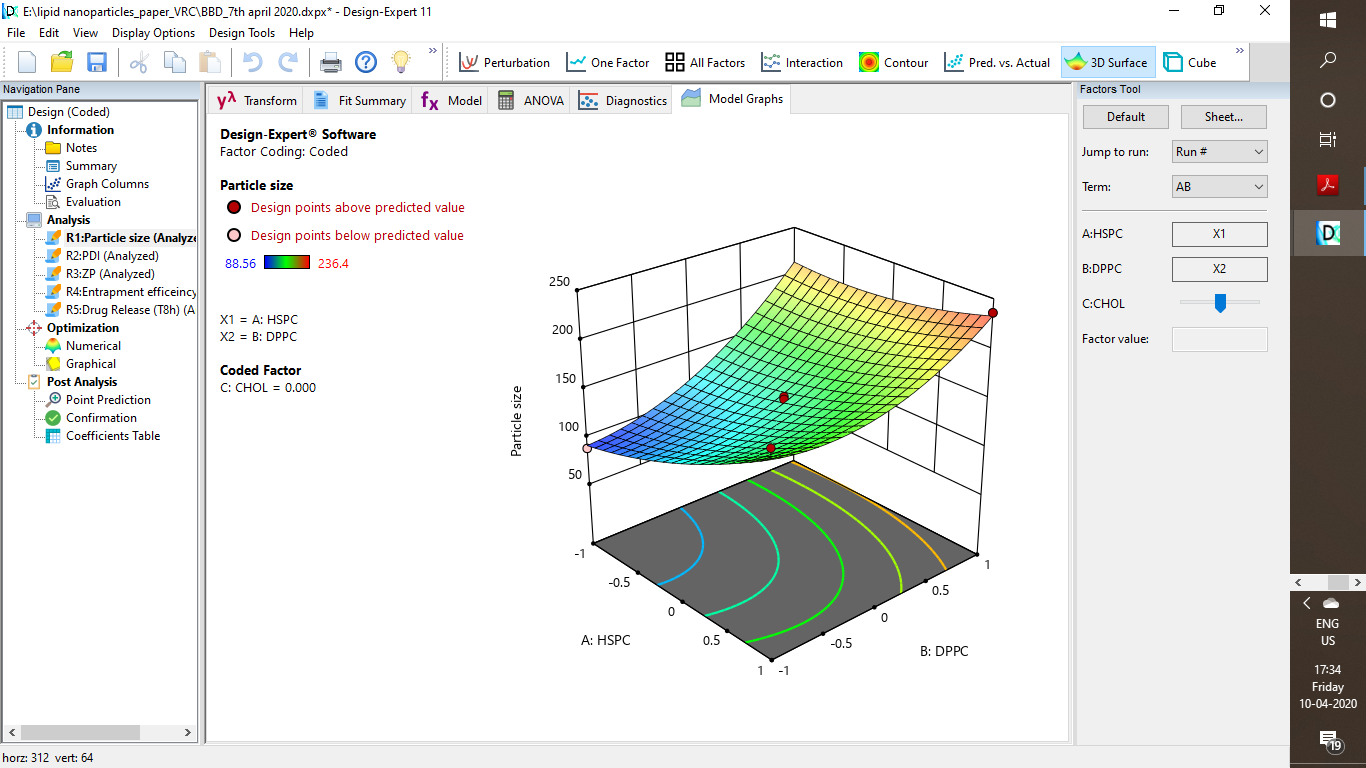

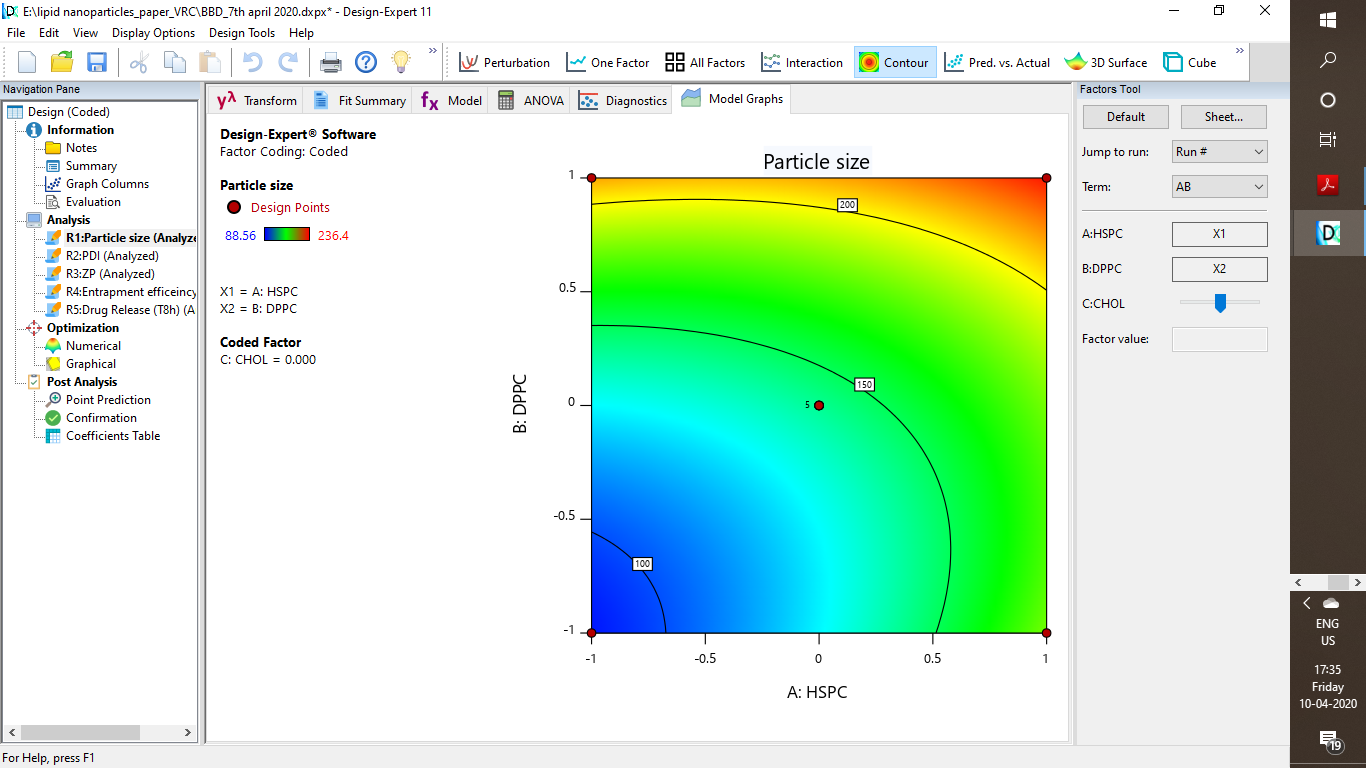


A(i) A(ii)


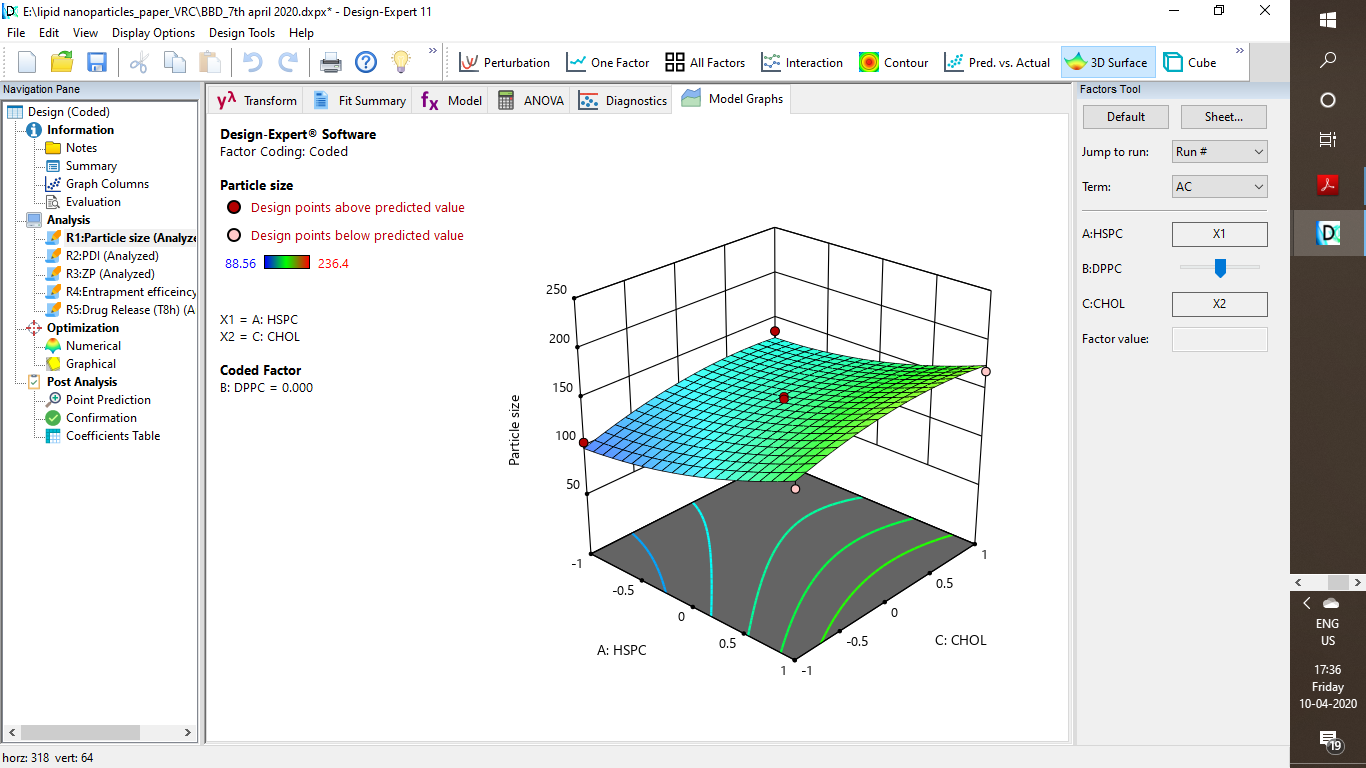

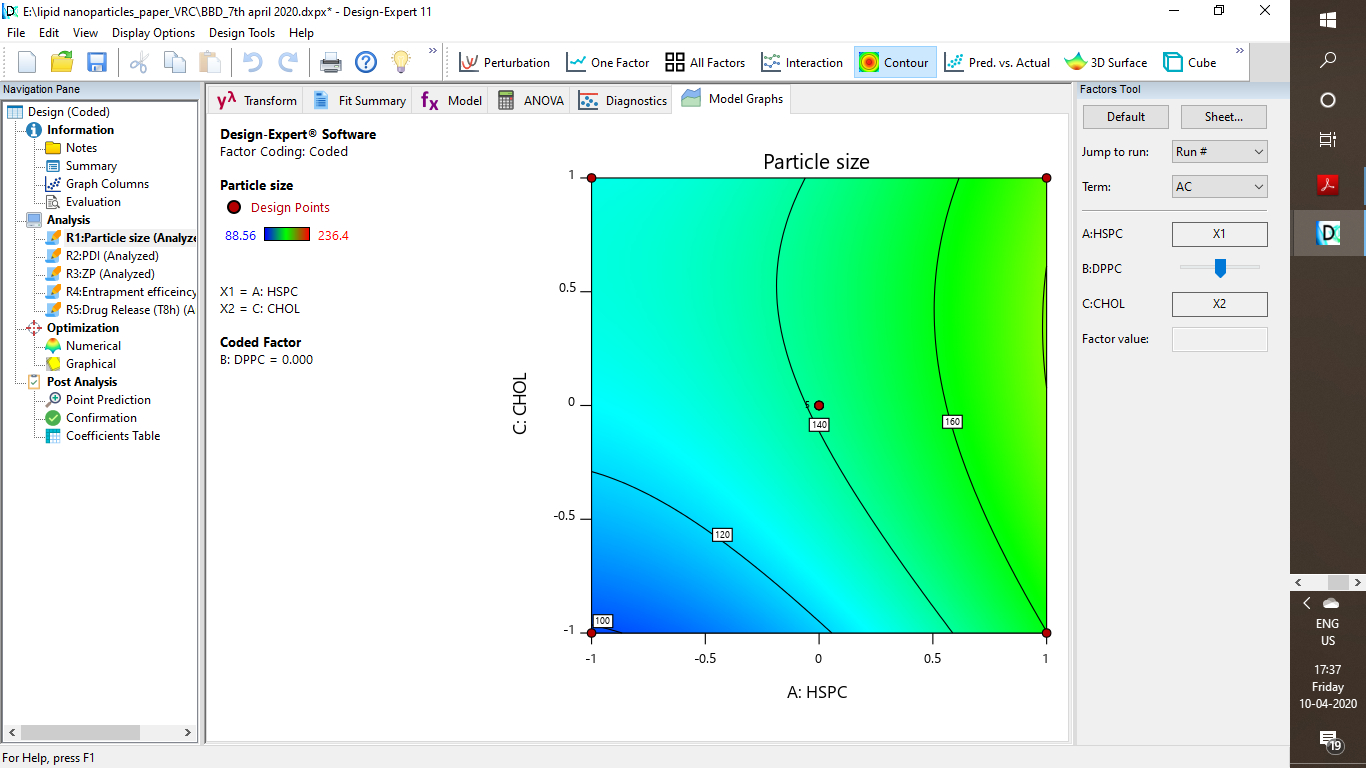


B(i) B(ii)


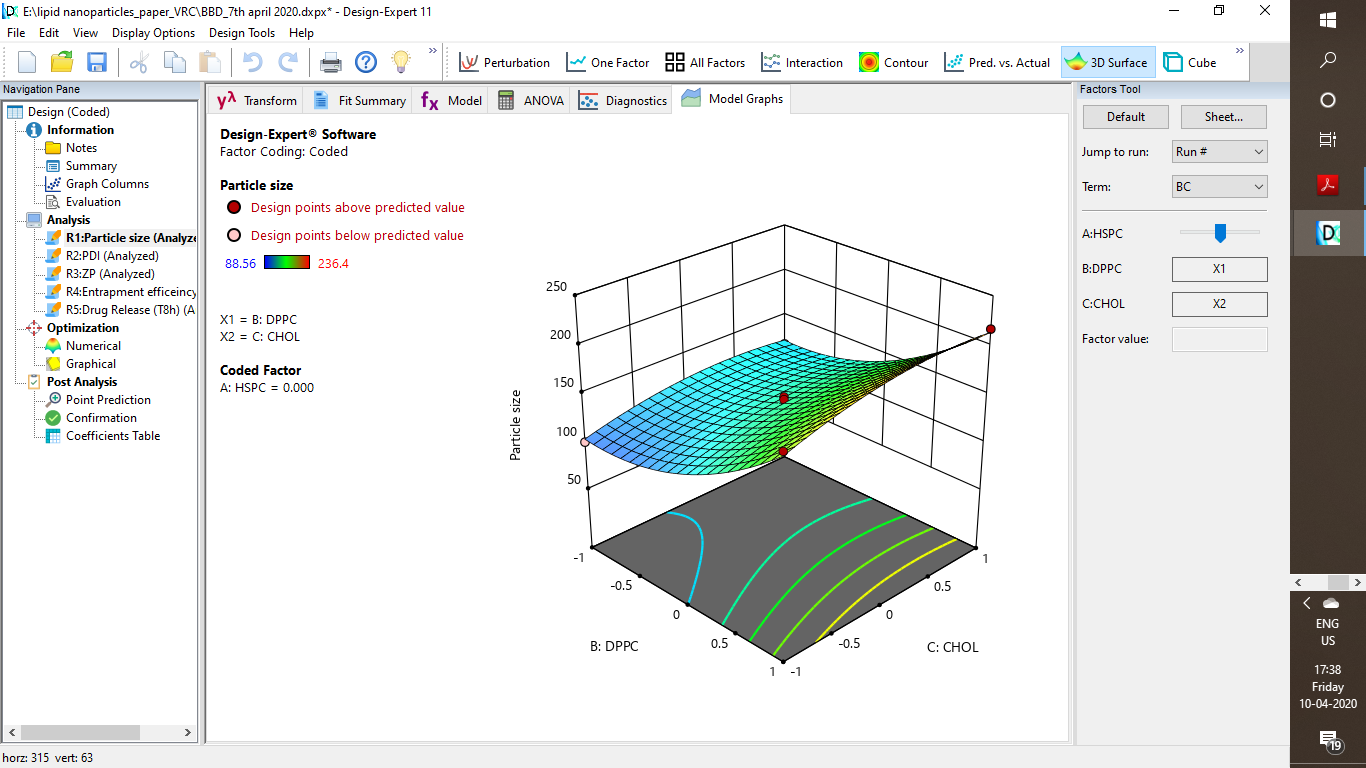

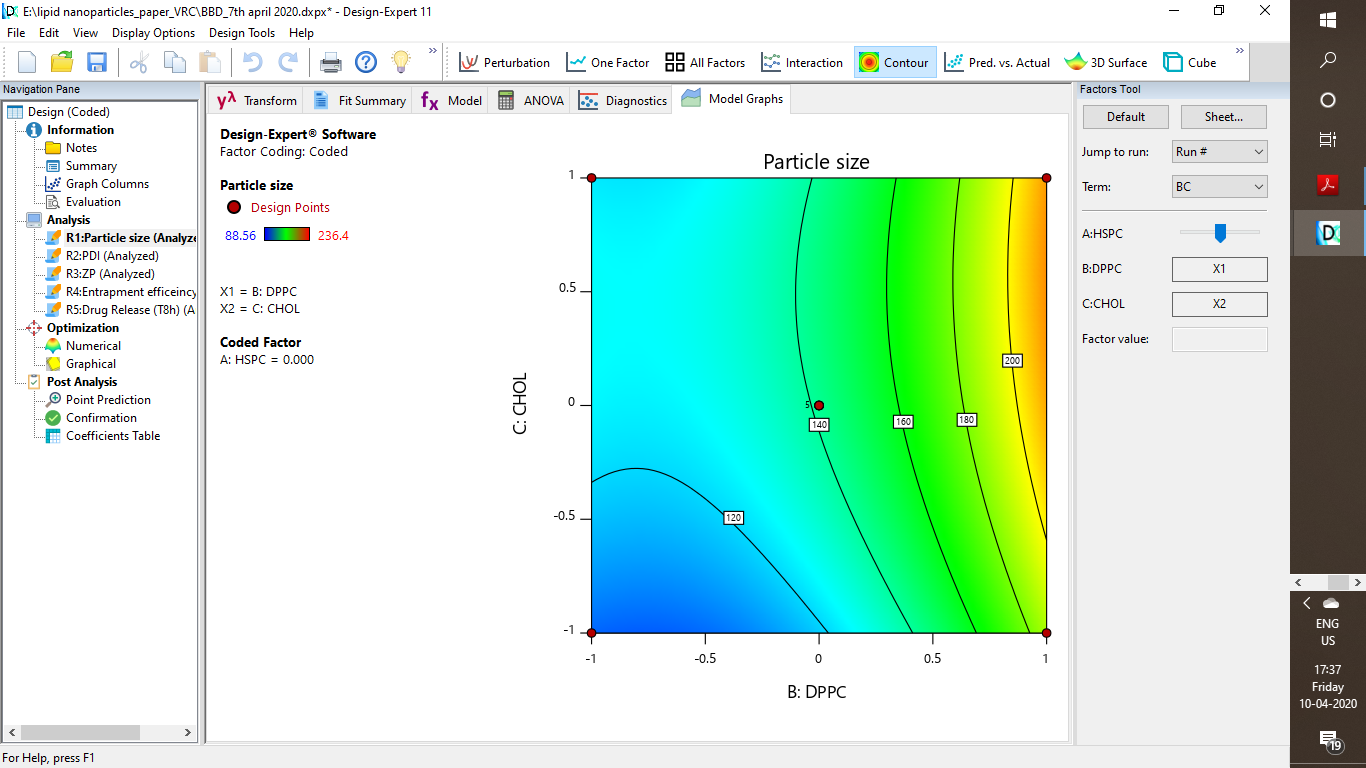


C(i) C(ii)

**Figure S2 (A-C):** 3D-response surfaces and their 2D-contour plots depicting the influence of CMAs on particle size of lipid nanovesicles of voriconazole


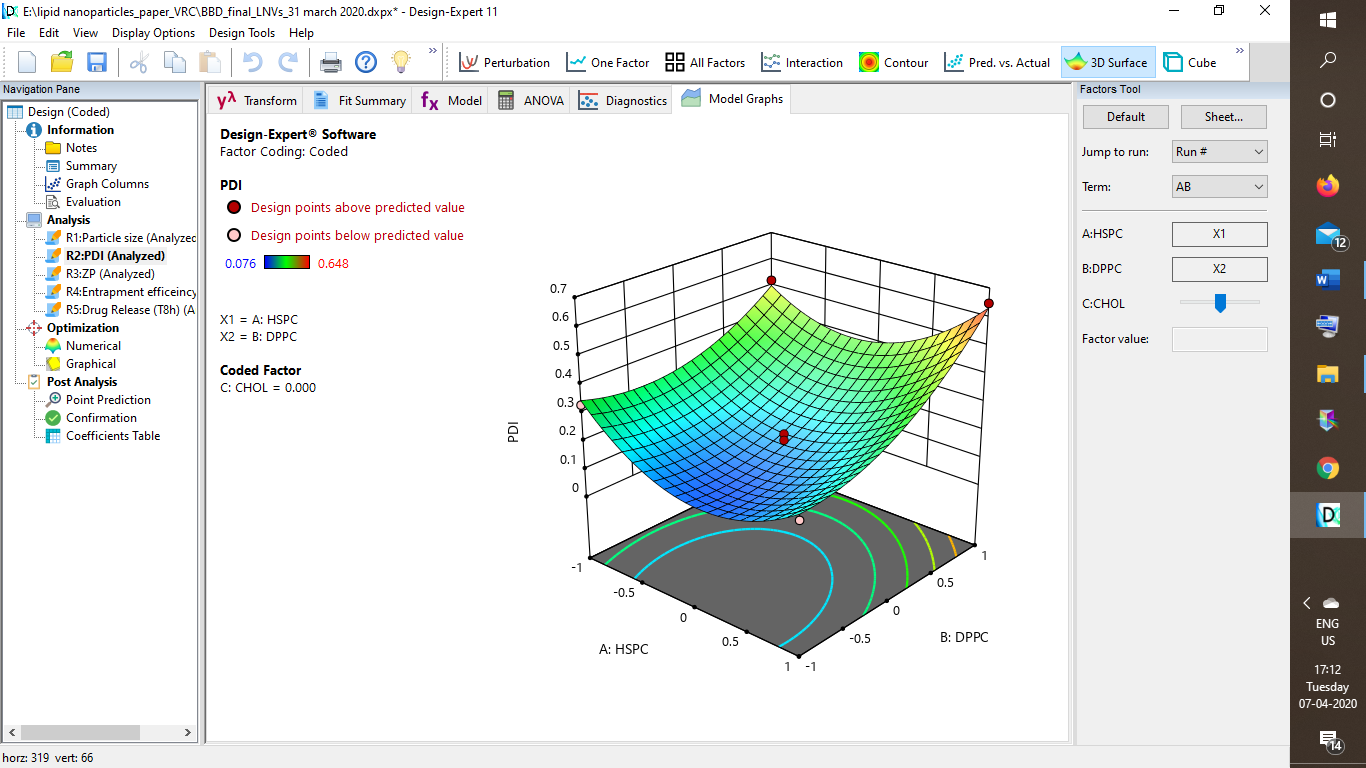

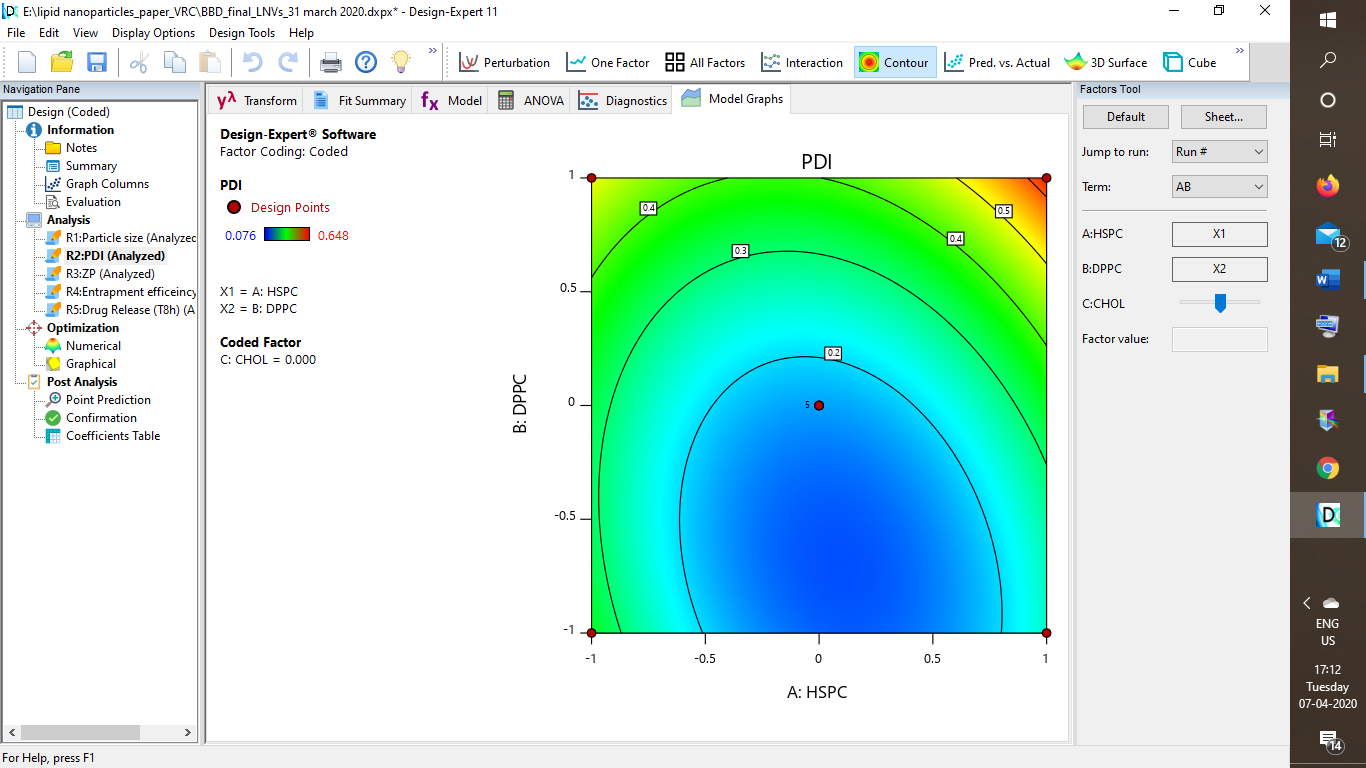


D(i) D(ii)


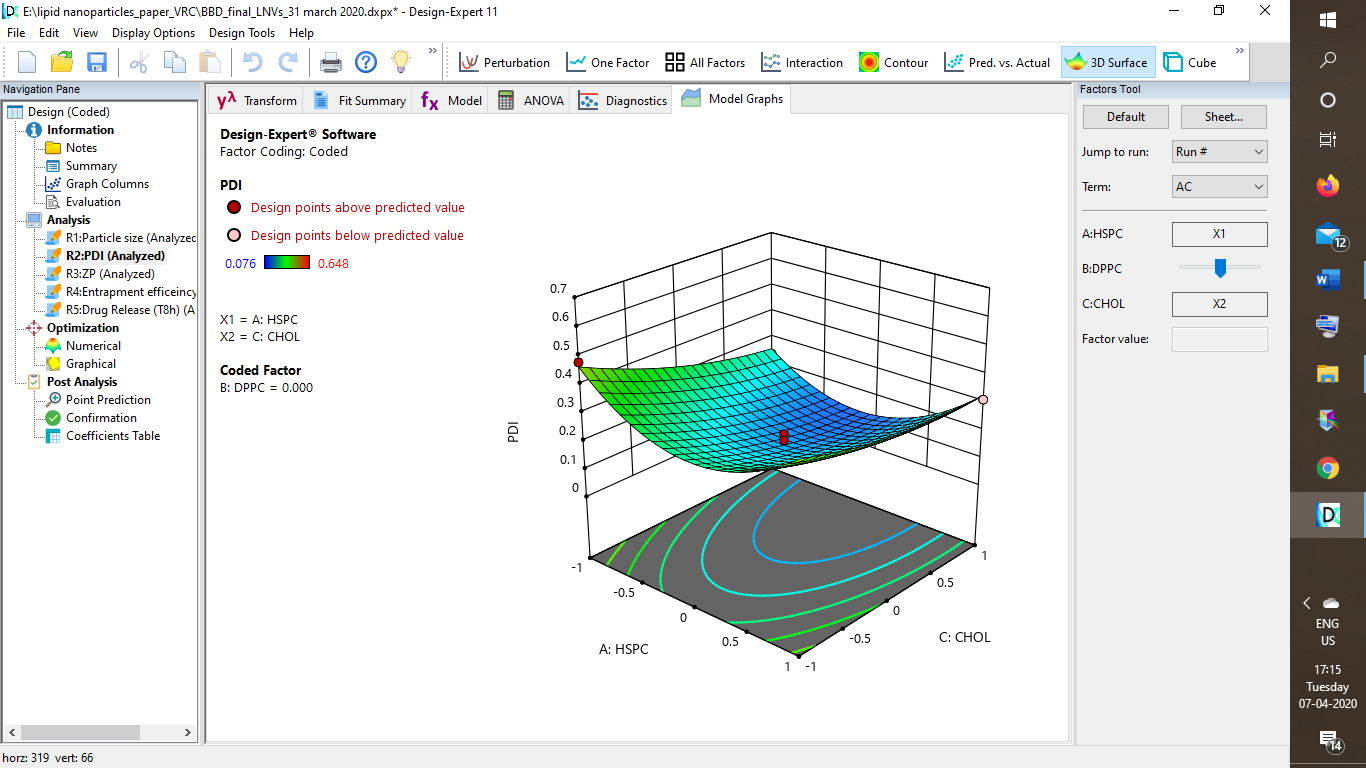

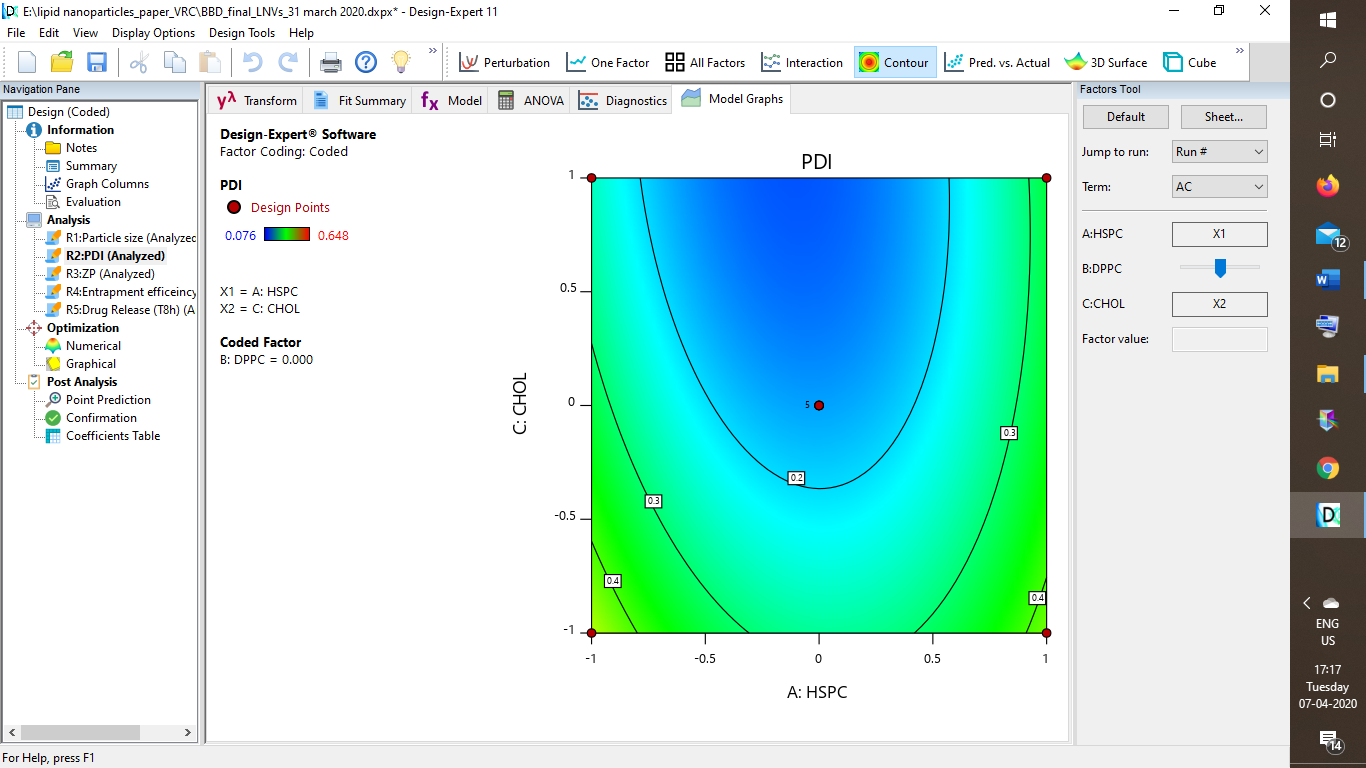


E(i) E(ii)


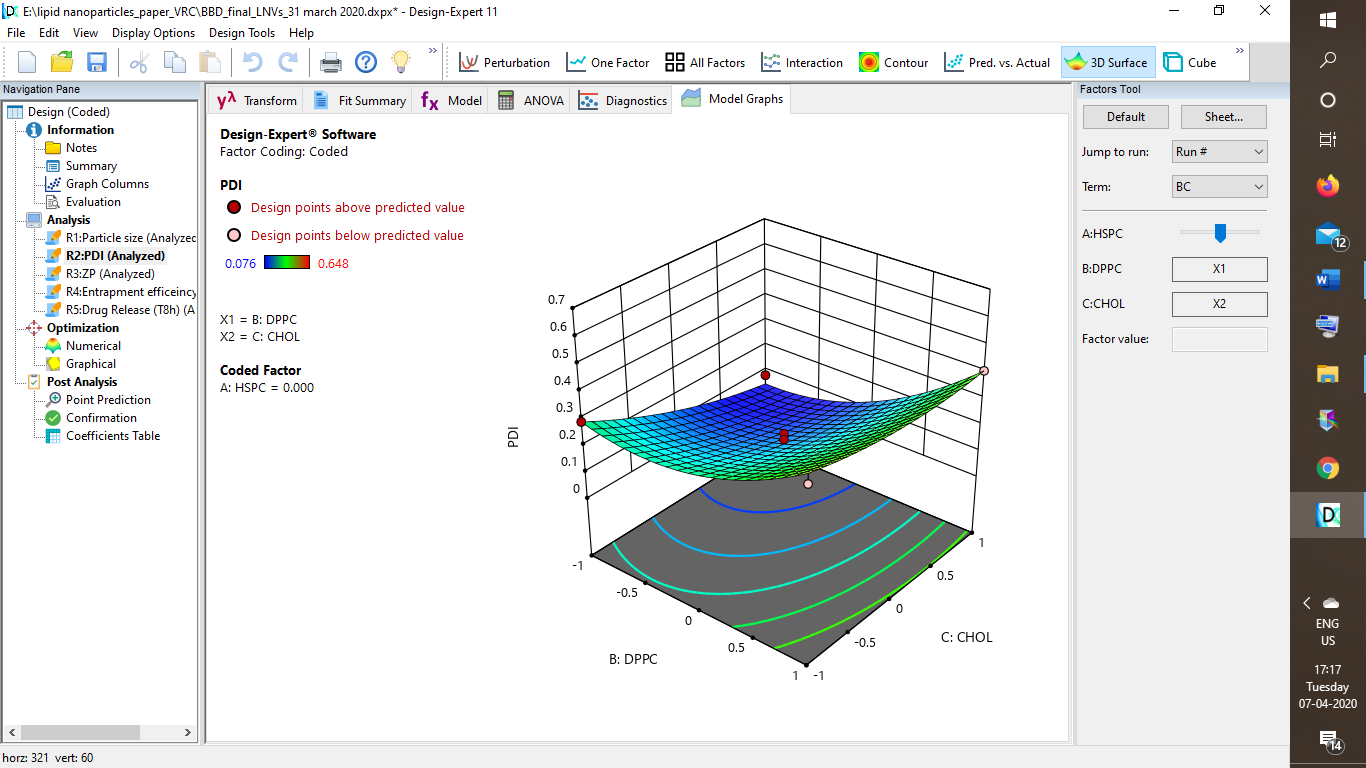

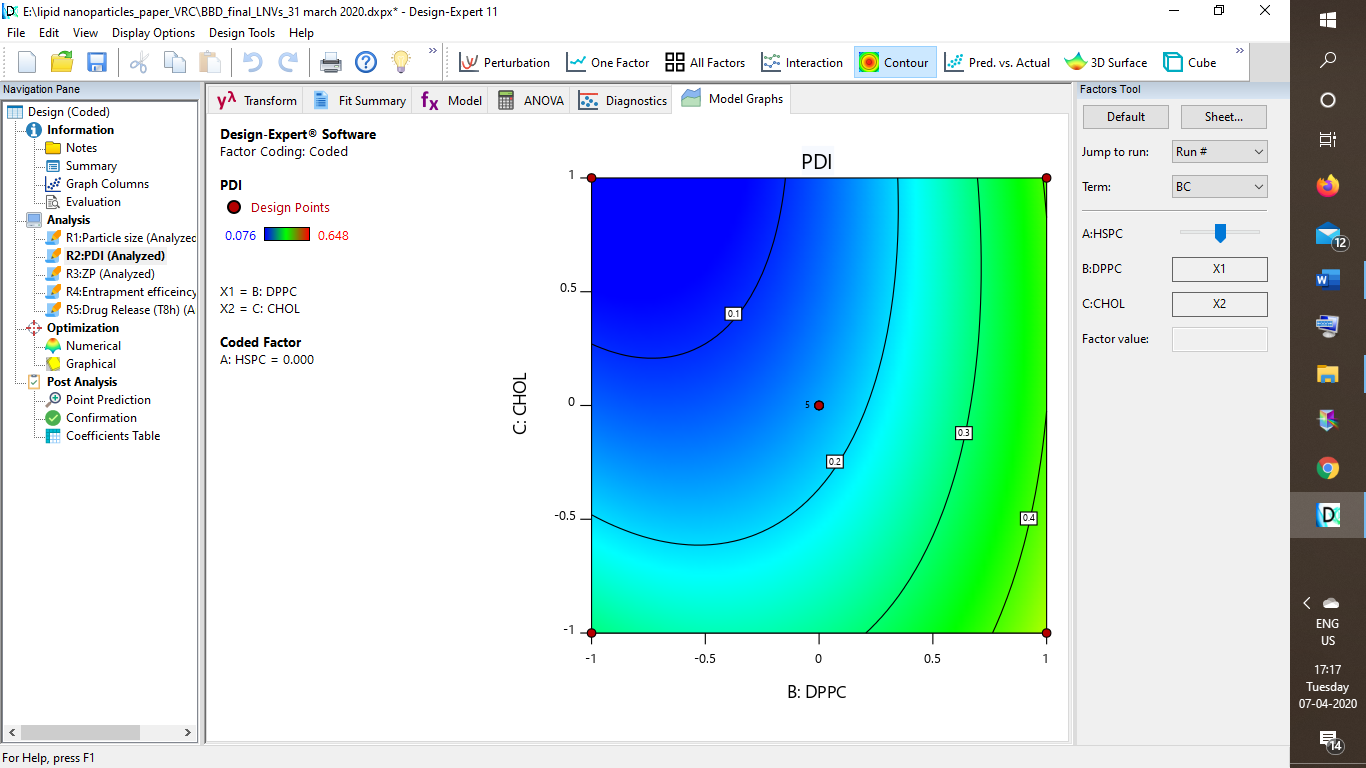


F(i) F(ii)

**Figure S2 (D-F):** 3D-response surfaces and 2D-contour plots depicting the influence of CMAs on polydispersity index of lipid nanovesicles of voriconazole


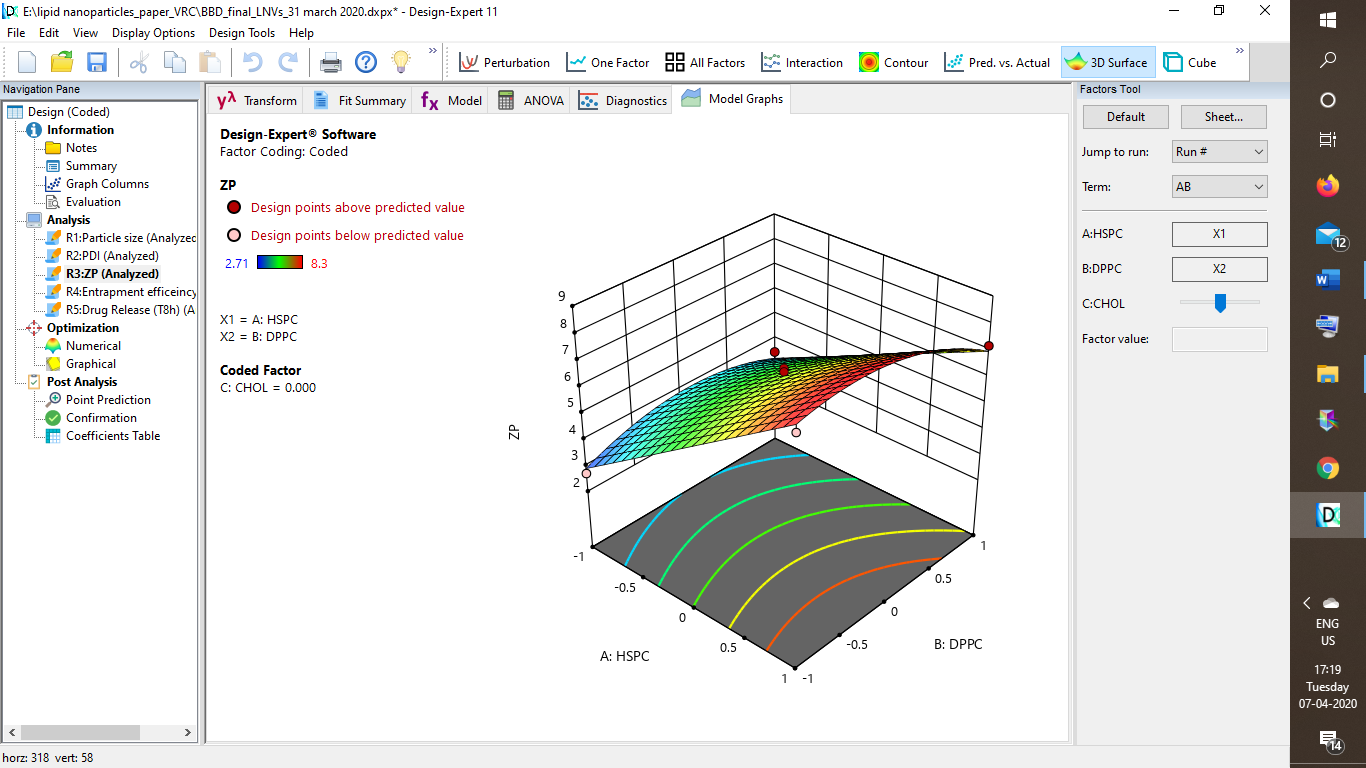

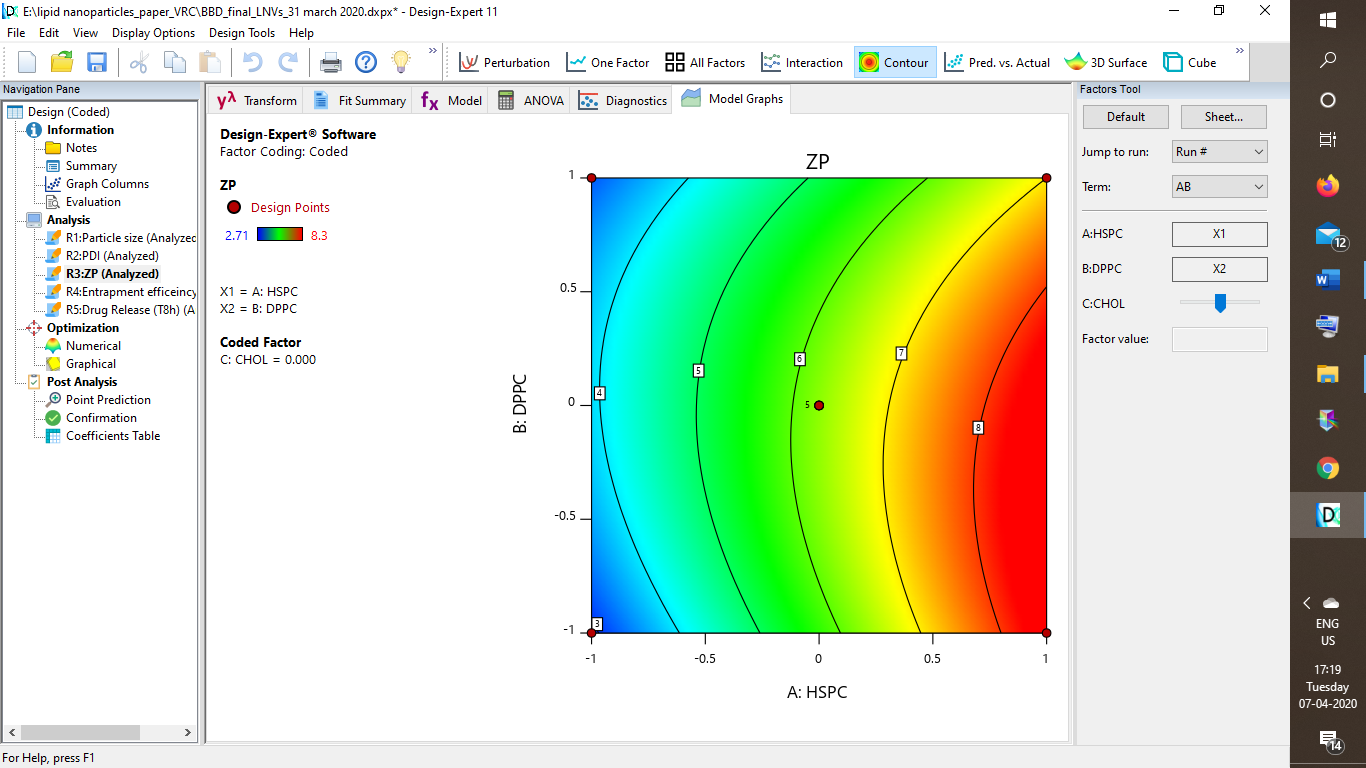


G(i) G(ii)


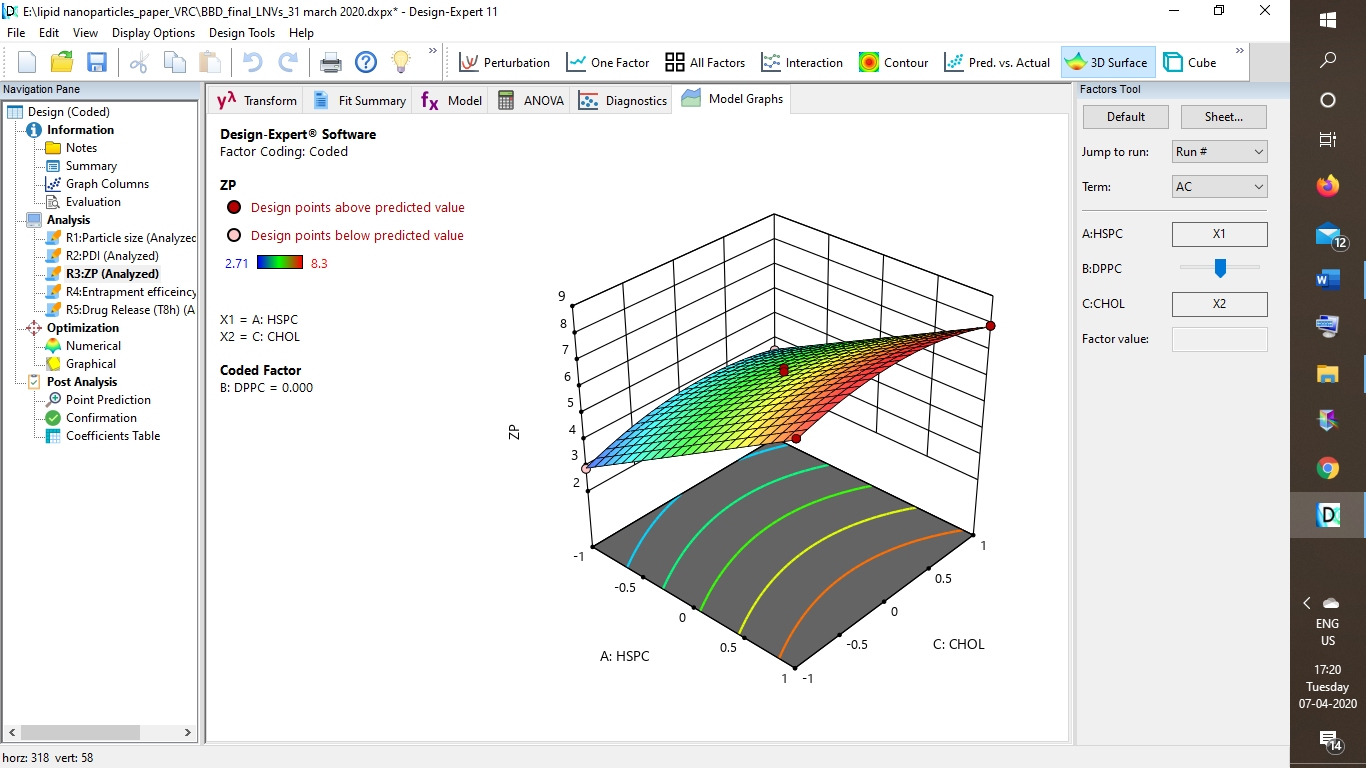

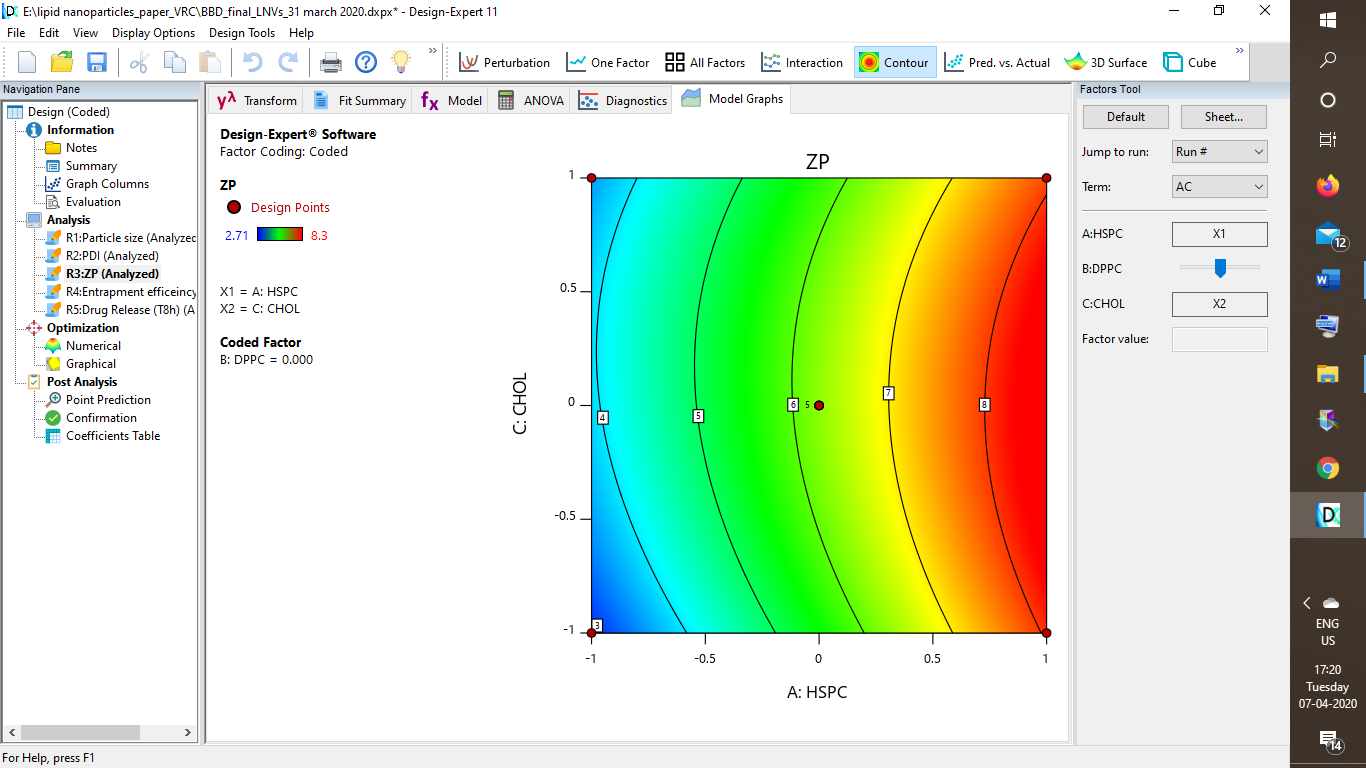


H(i) H(ii)


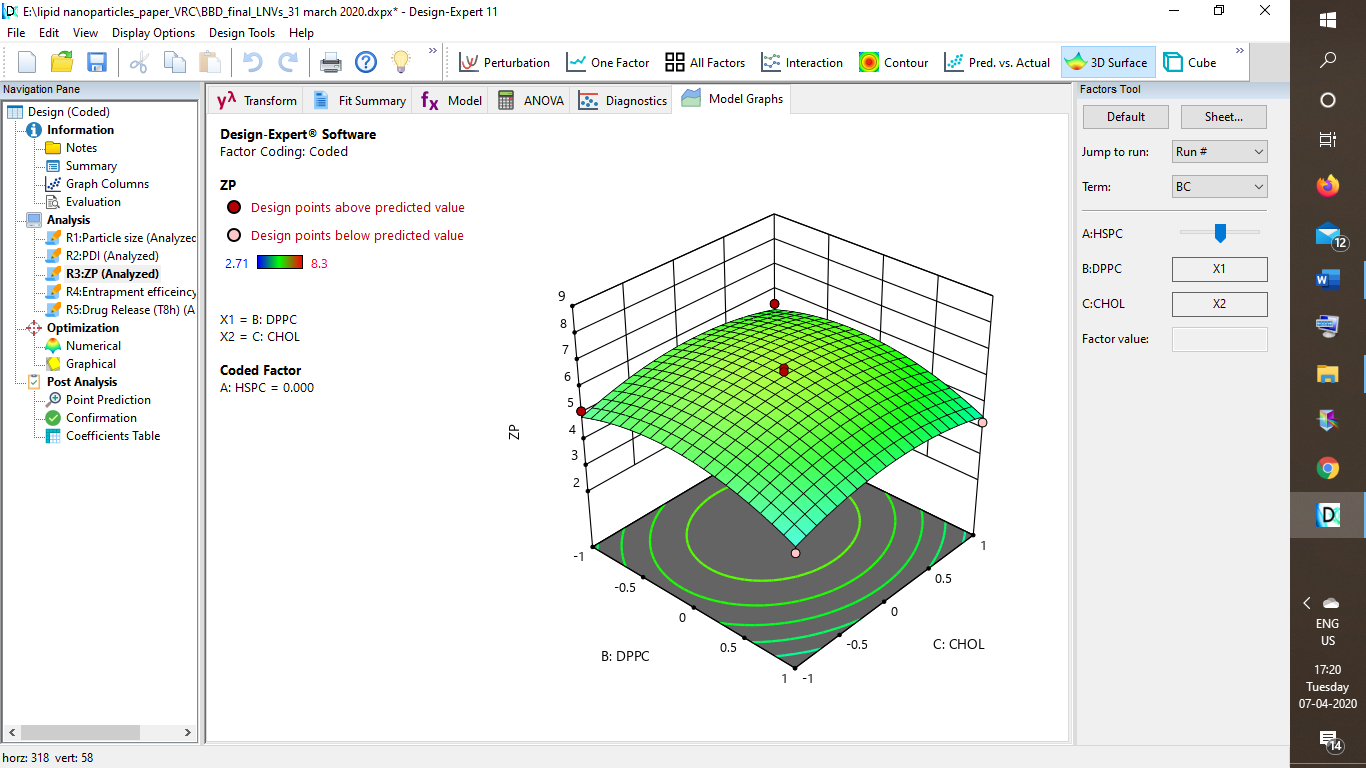

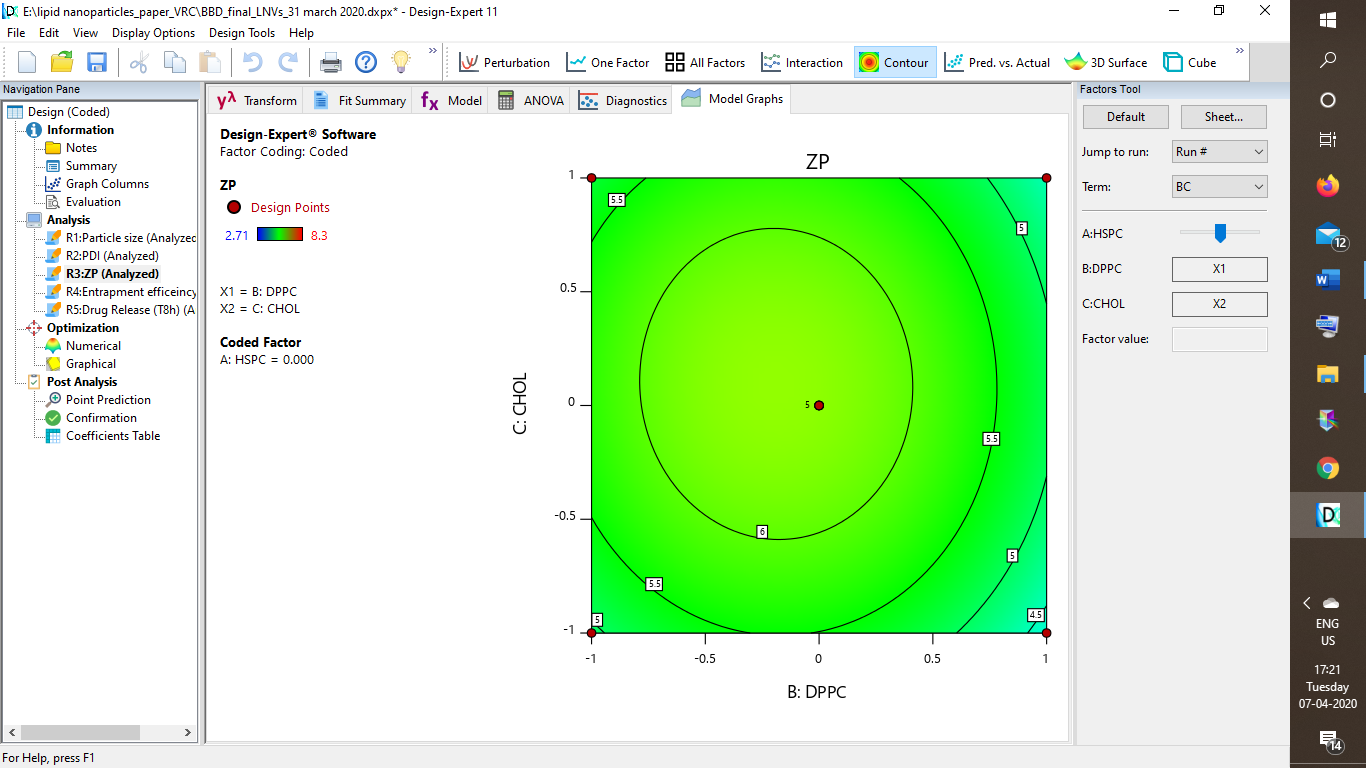


I(i) I(ii)

**Figure S2 (G-I):** 3D-response surfaces and 2D-contour plots depicting the influence of CMAs on zeta potential of lipid nanovesicles of voriconazole


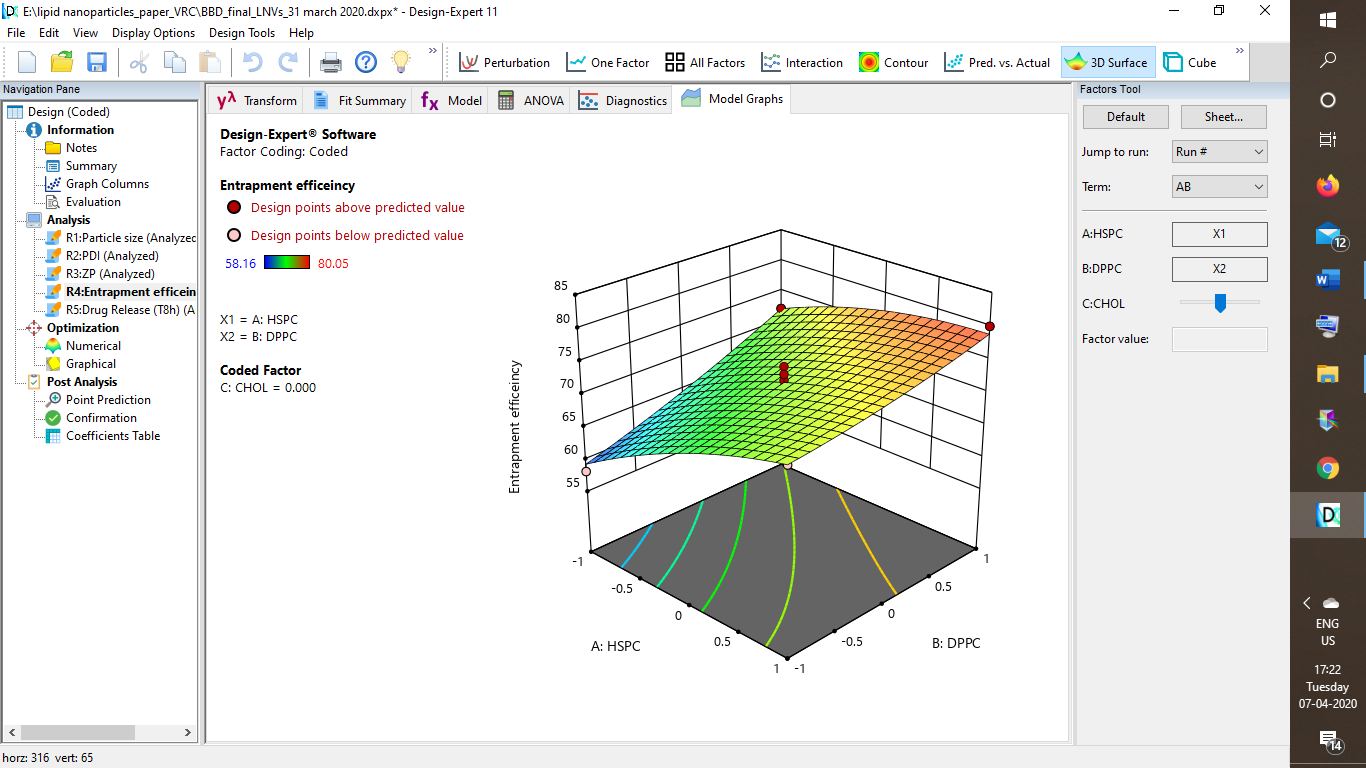

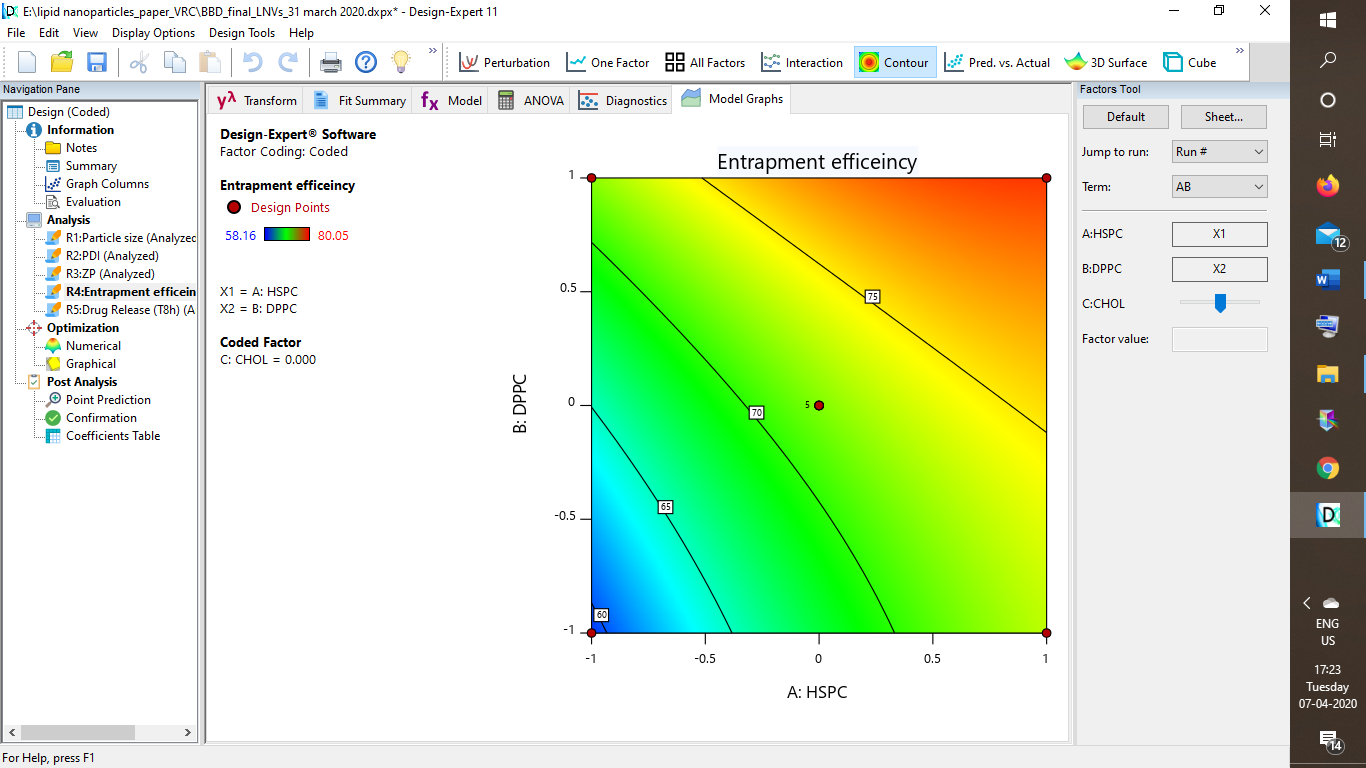


J(i) J(ii)


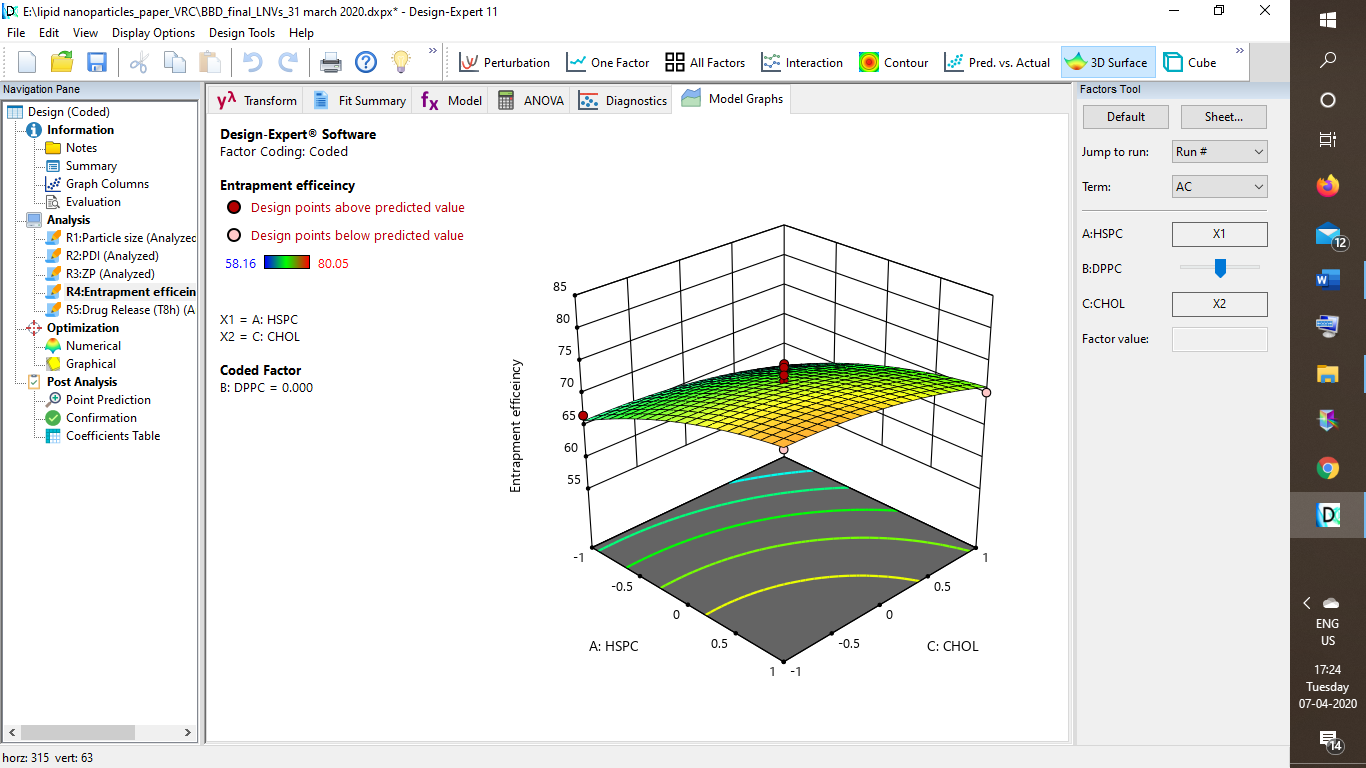

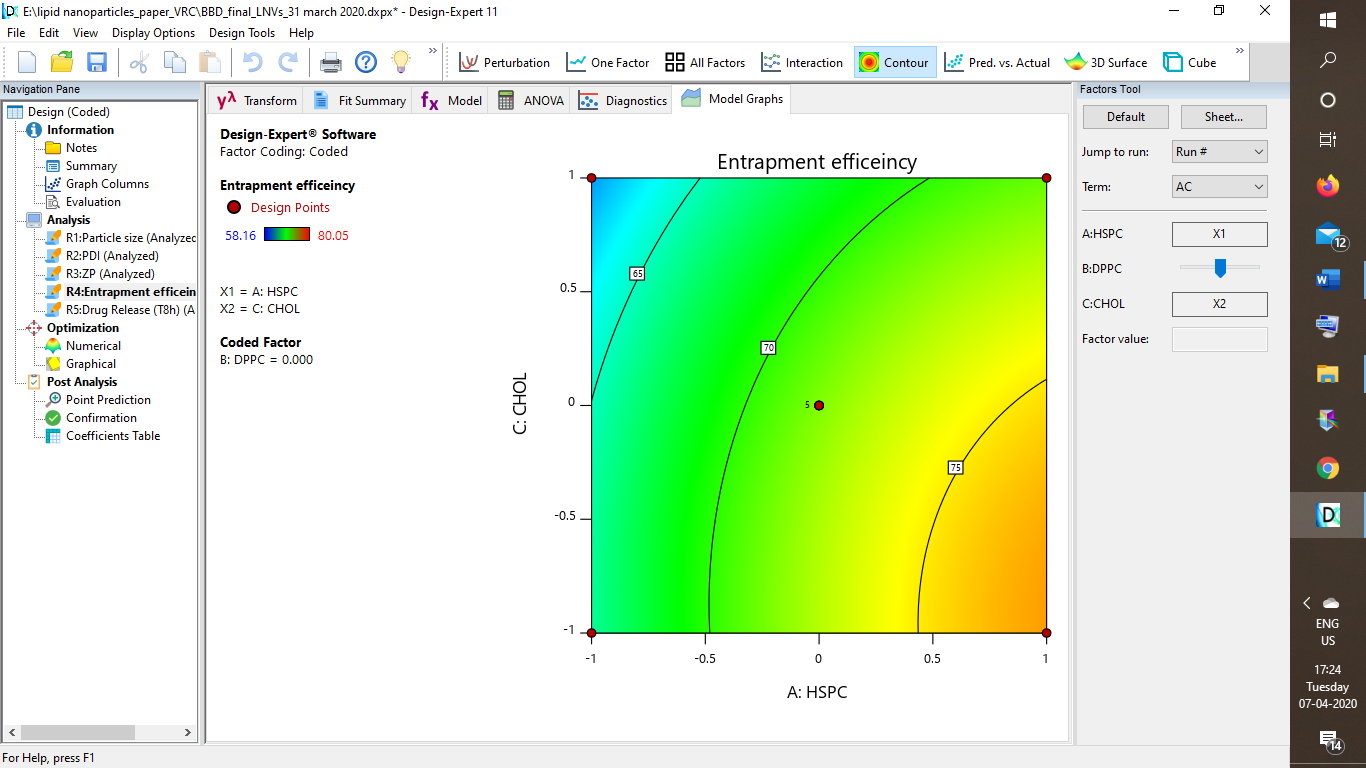


K(i) K(ii)


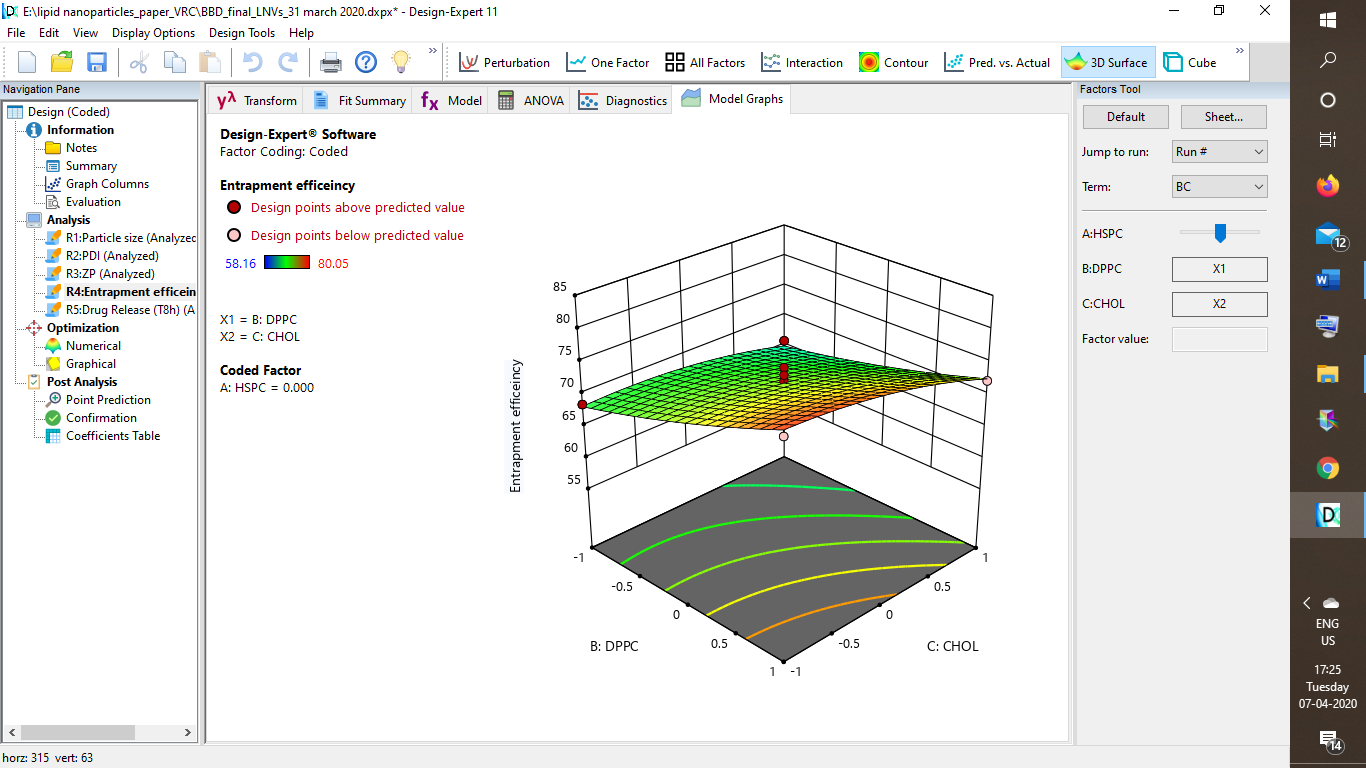

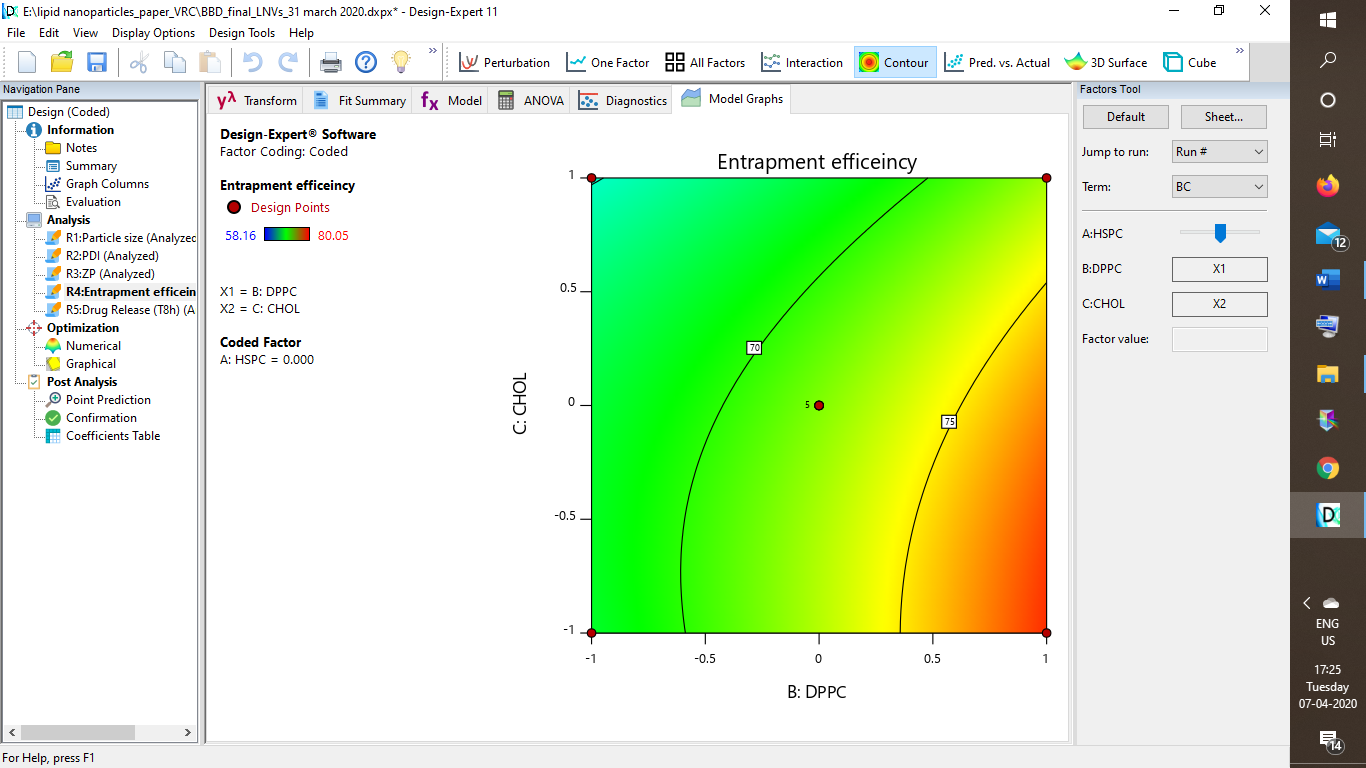


L(i) L(ii)

**Figure S2 (J-L):** 3D-response surfaces and 2D-contour plots depicting the influence of CMAs on entrapment efficiency of lipid nanovesicles of voriconazole


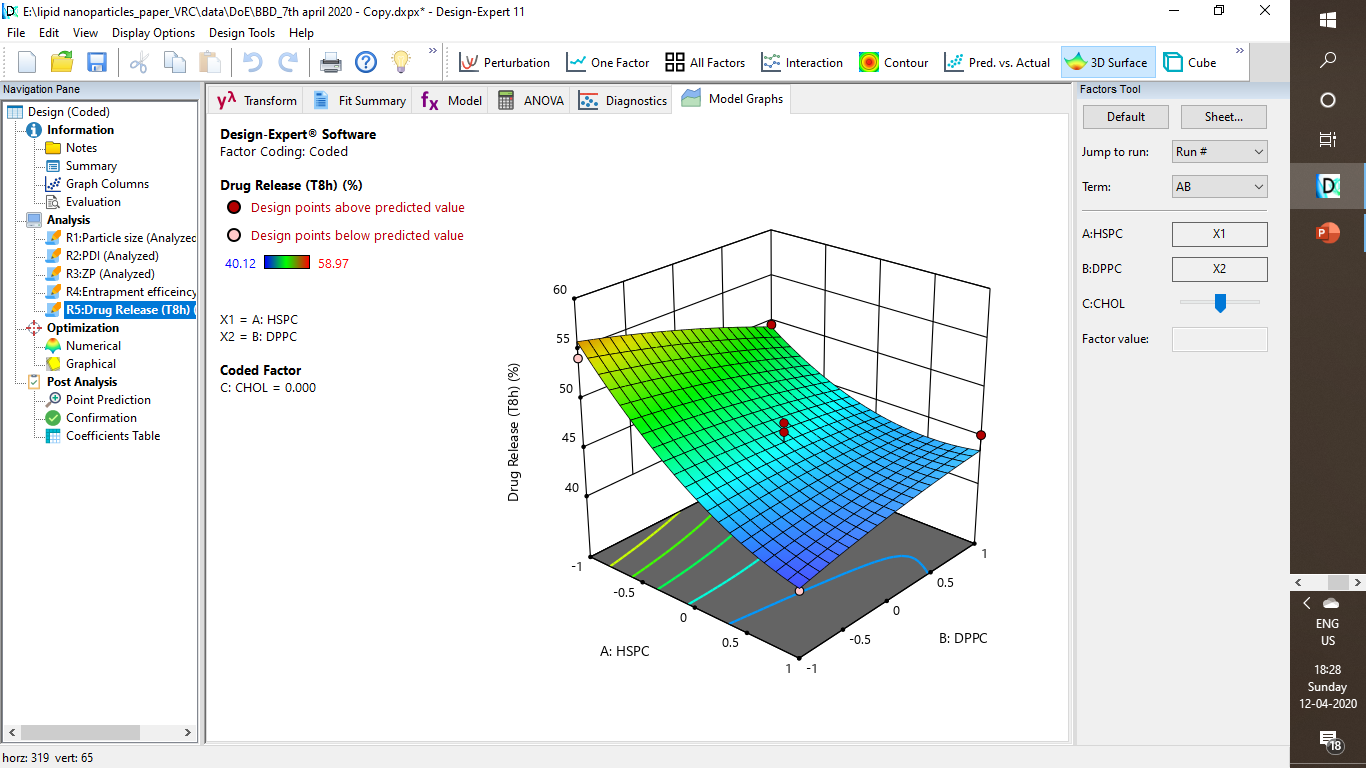

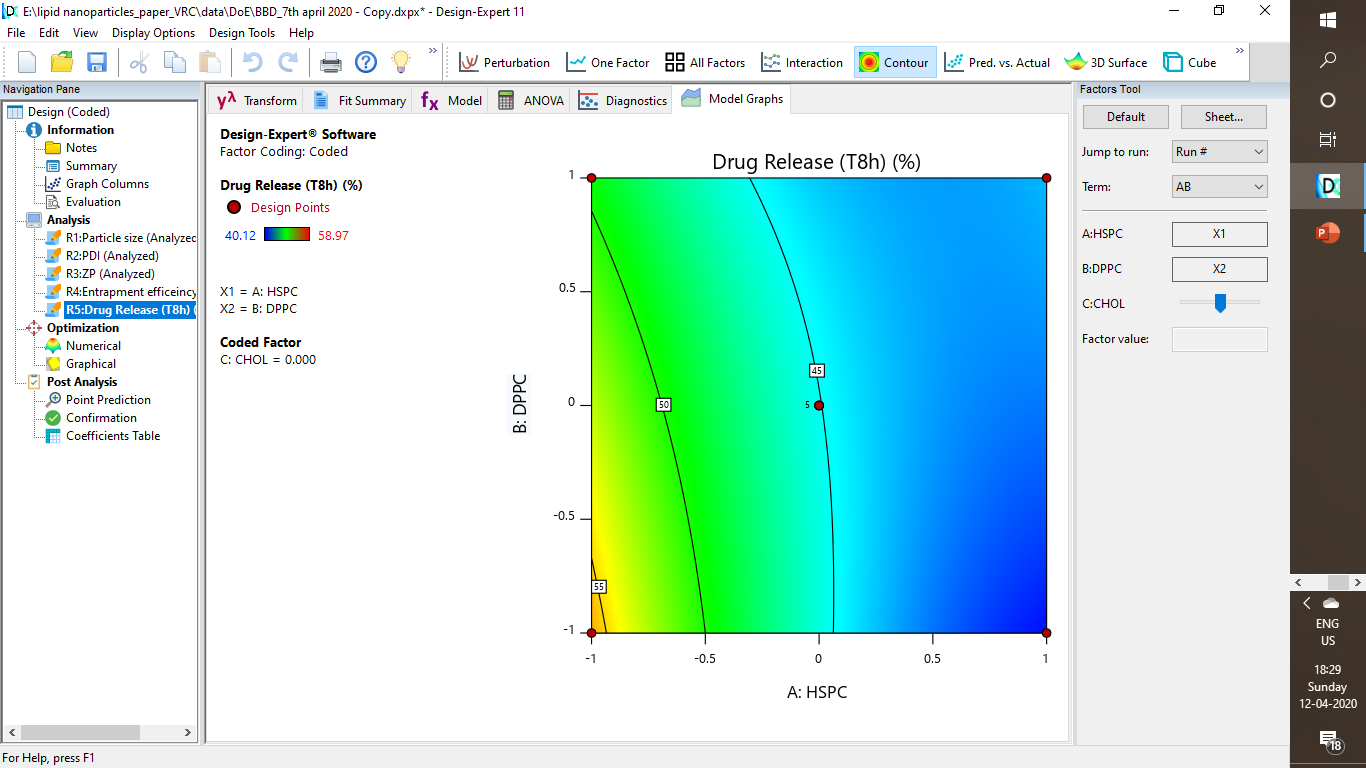


M(i) M(ii)


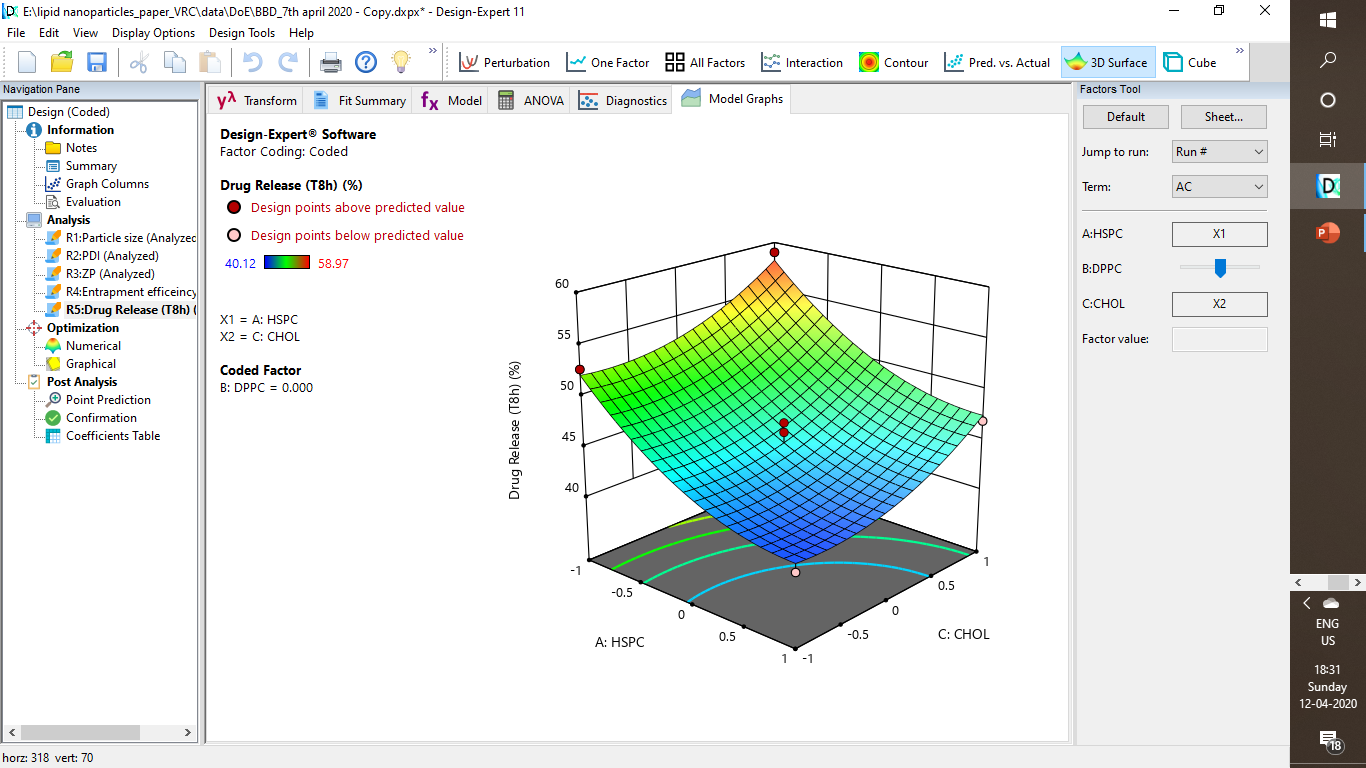

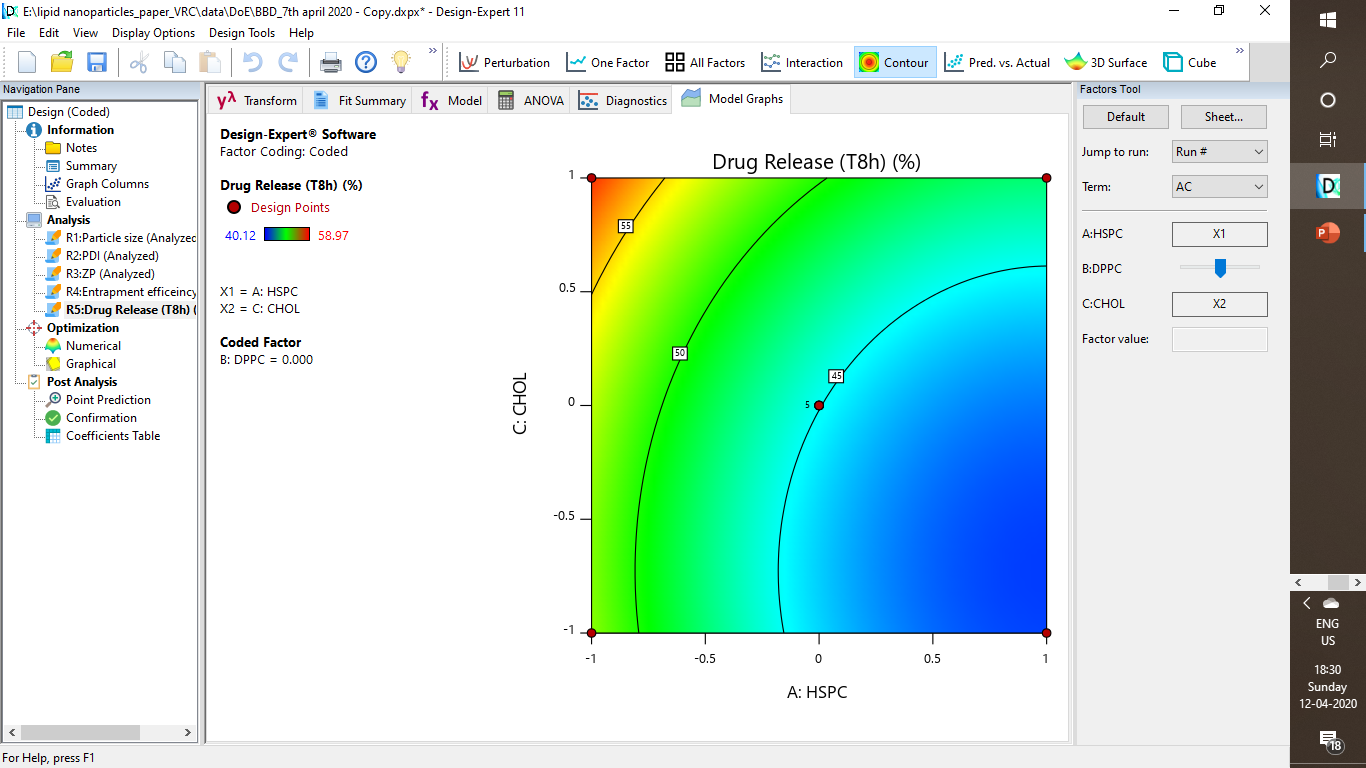


N(i) N(ii)


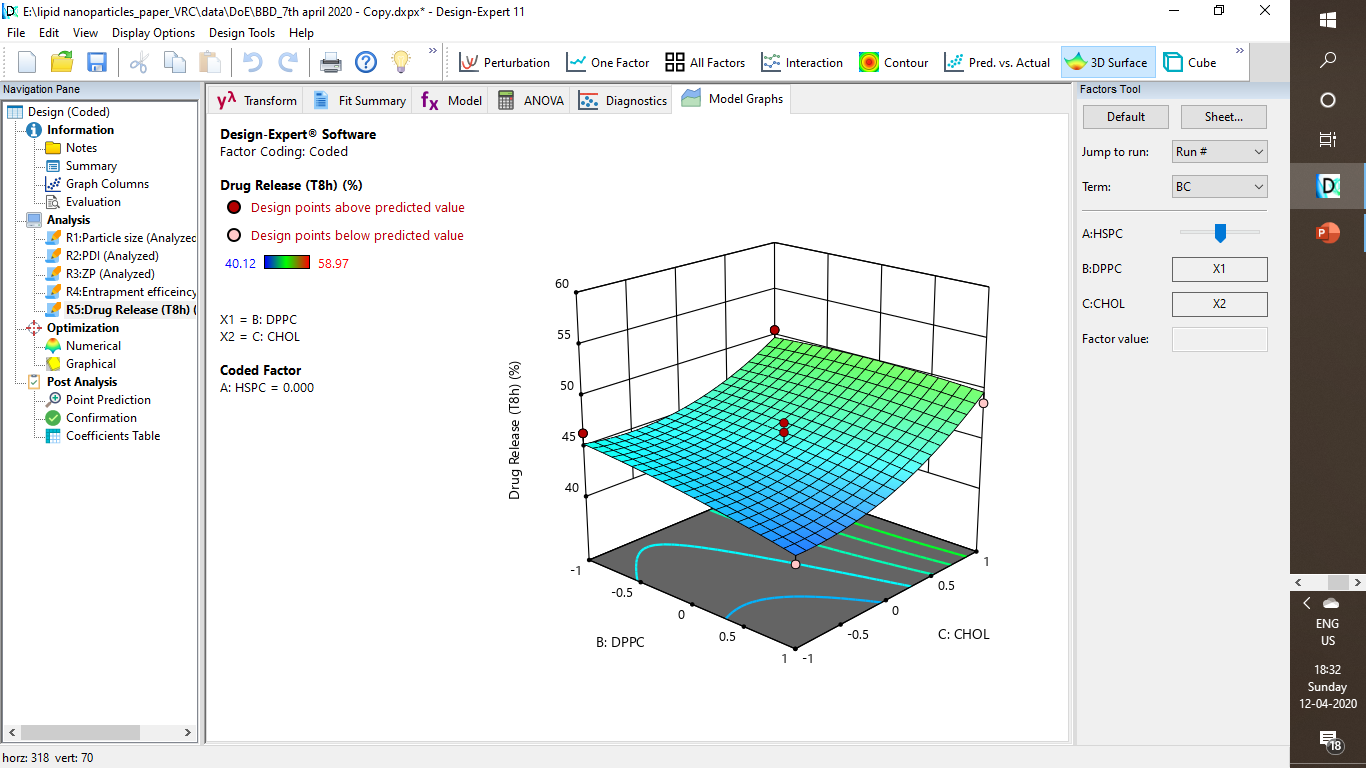

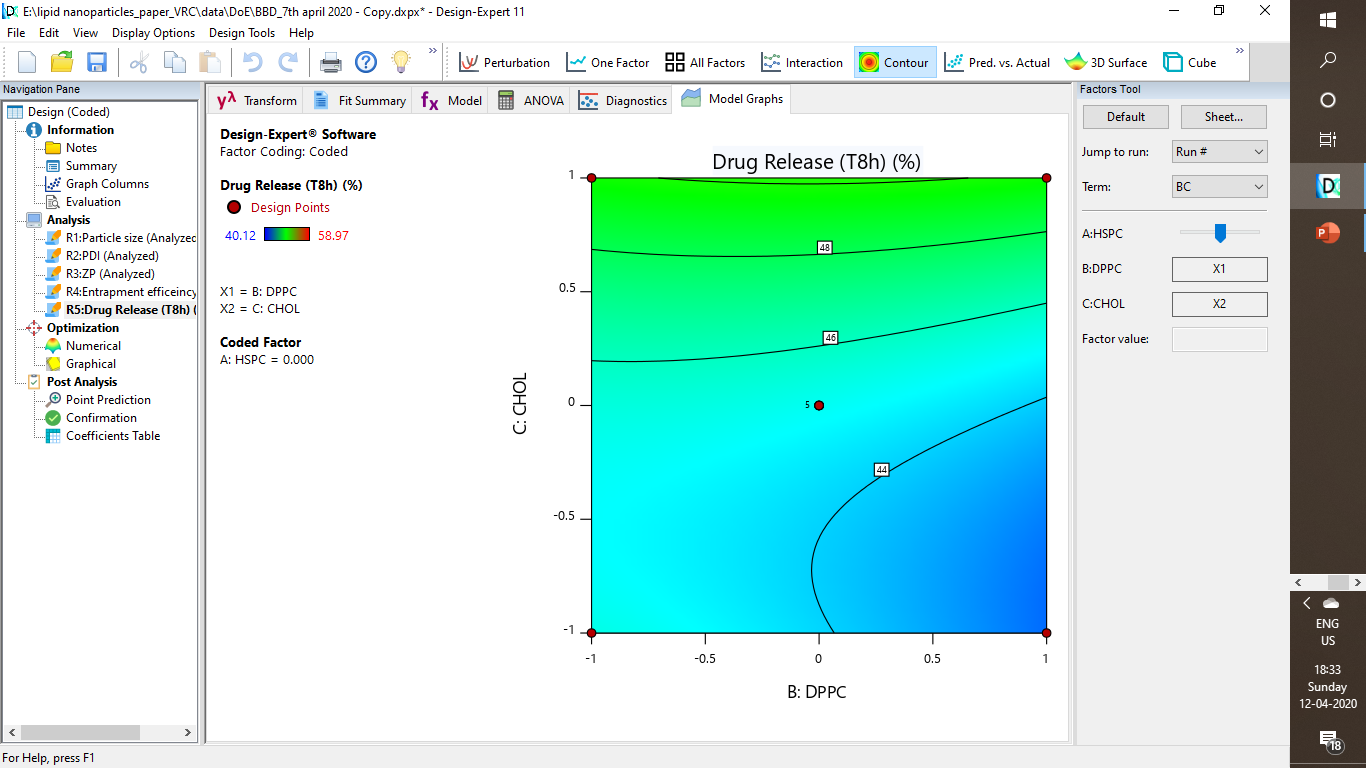


O(i) O(ii)

**Figure S2 (M-O):** 3D-response surfaces and 2D-contour plots depicting the influence of CMAs on drug release of at 8h


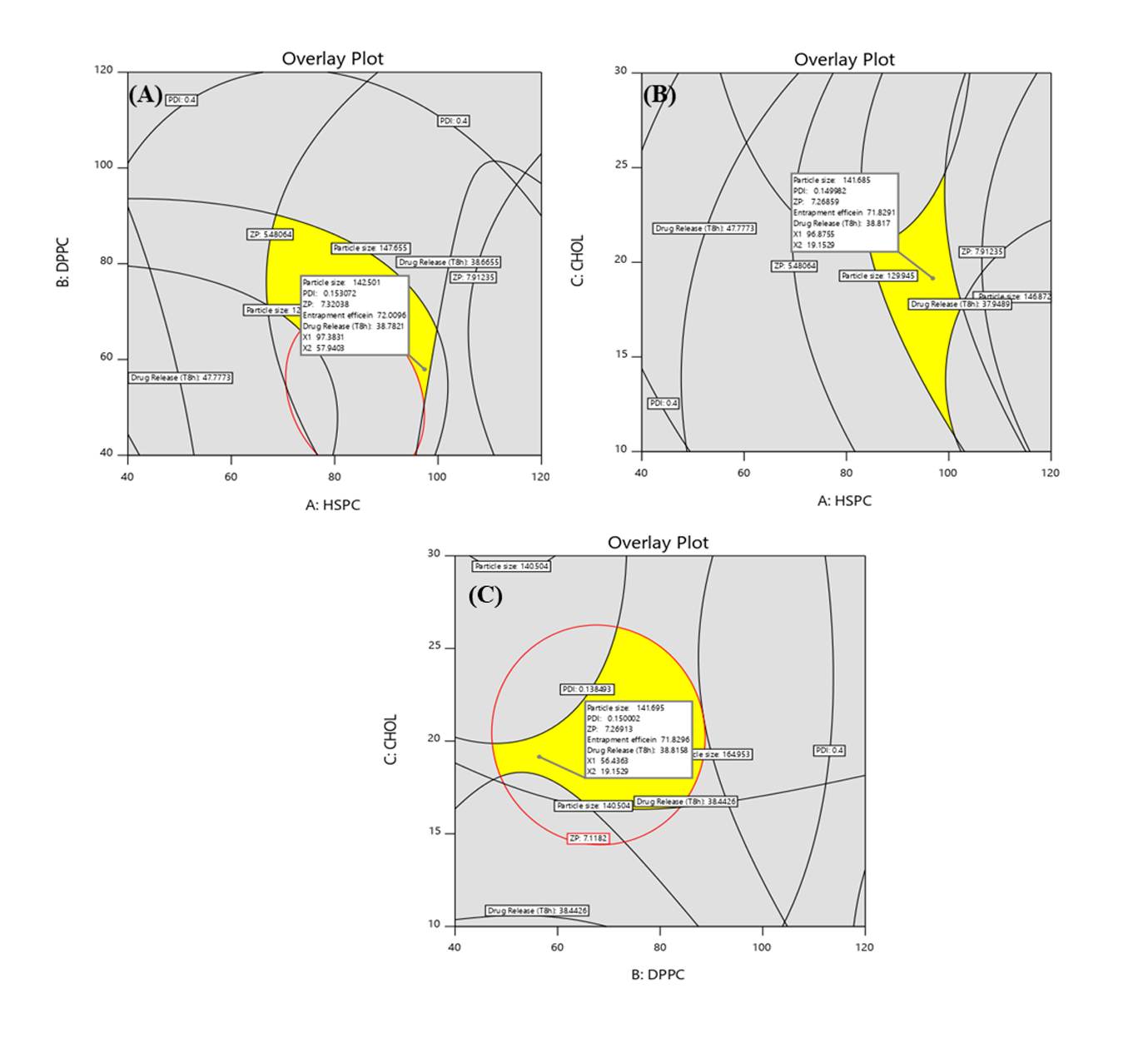


**Figure S3**: Overlay plots depicting the design space and demarcation of optimised lipid nanovesicles of voriconazole


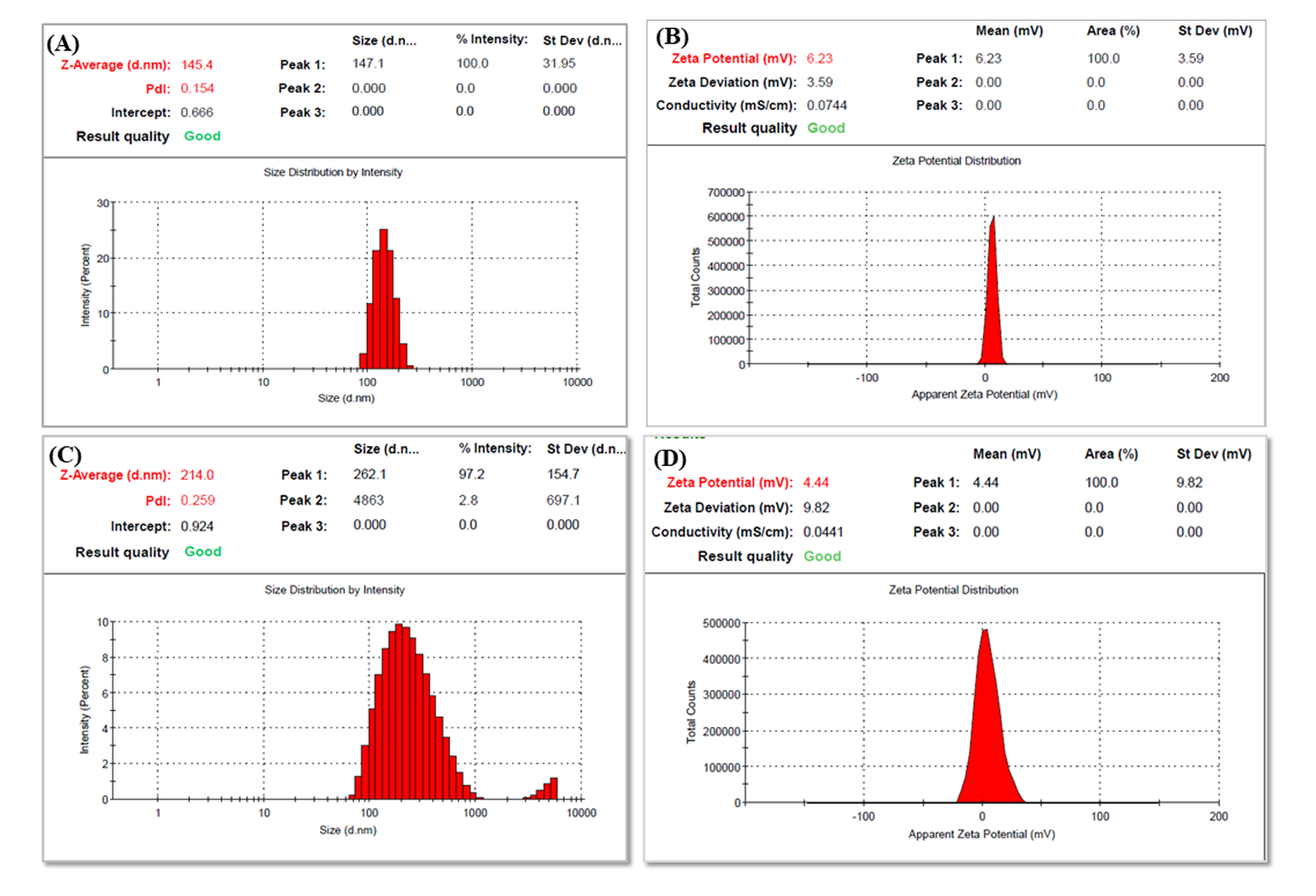


**Figure S4:** Globule size distribution and zeta potential of optimized lipid nanovesicles (LNVs) in water (A and B) and PBS (C and D)


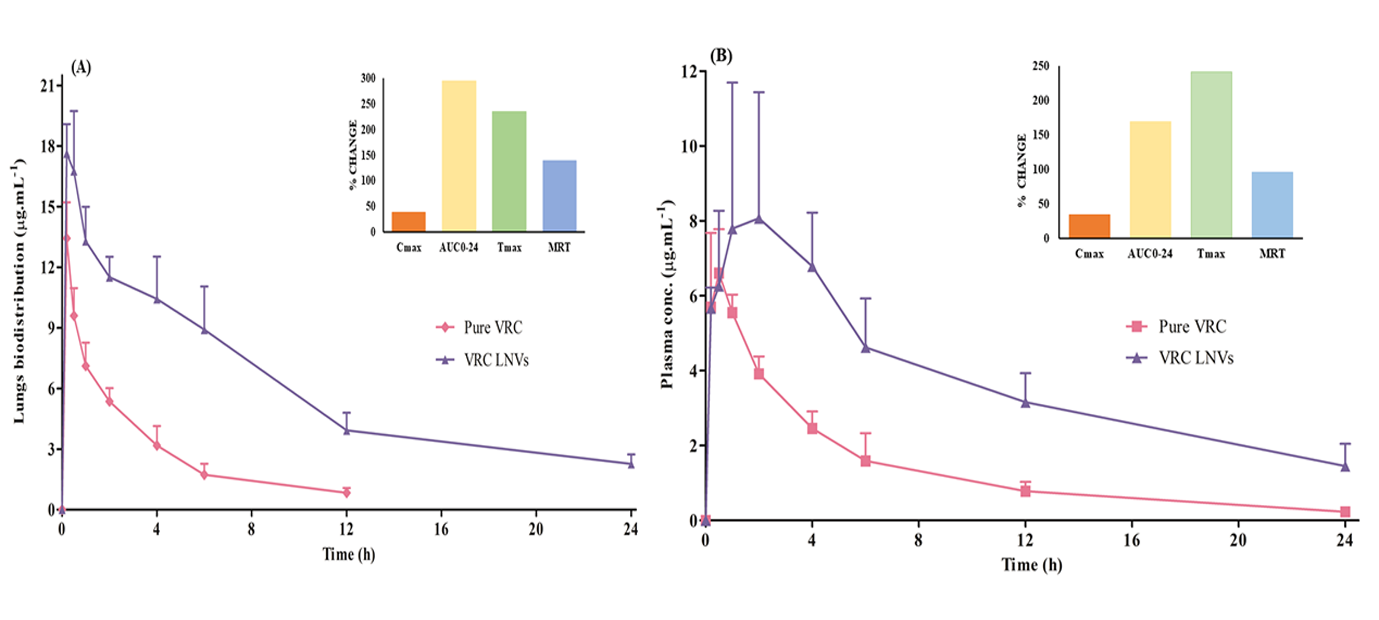


**Figure S5:** Pharmacokinetic profiles in Balb/c mice of lipid nanovesicles (LNVs) of voriconazole (VRC) and pure VRC. (A) Lungs and (B) Plasma. The corresponding inset portrays the propionate change in the pharmacokinetic parameters. Data represent Mean± SD (n=3).


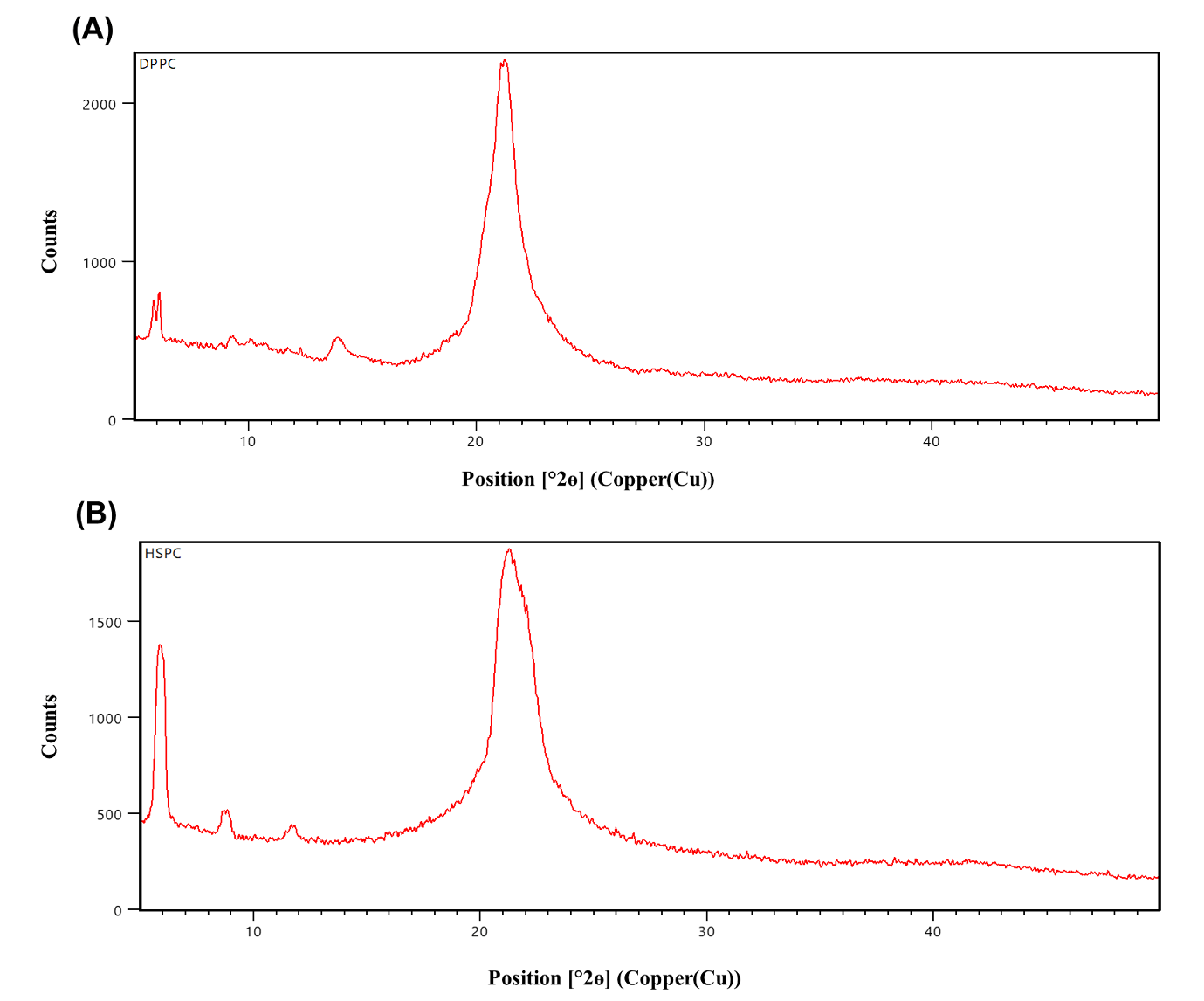


**Figure S6:** P-XRD diffractogram of (A) dipalmitoylphosphatidylcholine (DPPC) and (B) hydrogenated soya-phosphatidylcholine (HSPC)

*Formulation development employing DoE*

*Optimization Studies*

Response surface mapping assisted in facilitating the dependence, inter-dependence and co-variation among the studied variables. The polynomial coefficient analysis was carried out employing quadratic model (Supplementary **Equation S1**) using multiple linear regression analysis option of Design Expert software Ver. 11.0 (Stat-Ease, Inc., Minneapolis, USA)

y = 𝜷_𝟎_ + 𝜷_𝟏_𝑿_𝟏_ + 𝜷_𝟐_𝑿_𝟐_ + 𝜷_𝟑_𝑿_3_ + 𝜷_𝟒_𝑿_1_ X_2_ + 𝜷_𝟓_𝑿_1_ X_3_ + 𝜷_𝟔_𝑿_2_ X_3_ + 𝜷_𝟕_𝑿_𝟏_^2^ + 𝜷_𝟖_𝑿_𝟐_^𝟐^+ 𝜷_9_𝑿_3_^𝟐^ …. Equation S1

where, y = response variable, β_0_ = intercept, β_1_ to β_3_ = coefficients of the linear model terms, β_4_ to β_6_ = coefficients of interaction terms, and β_7_ to β_9_ = coefficients of quadratic model terms.

The consequent 3D-response surface plots along with their 2D-contour graphs are depicted in **Figures S2 (A-O).**

**Figure S2 A(i)** illustrates the 3D-response surface plot among the chosen CMPs, *viz.,* HSPC and DPPC on PS of LNVs. Modest to sharp curvilinear increase in PS of LNVs was observed as the amounts of both of the lipids, *i.e.,* DPPC and HSPC, were increased from low to high levels, the effect of former being prominent. The results are akin to the observations reported by Chen *et al*., (2013), *i.e.,* rise in the lipid (e.g., DPPC) levels tends to enhance the globule size of LNVs, prepared by thin film hydration method [^1^](#_ENREF_1). Further, incorporation of CHL within the phospholipid bilayer also tends to modestly increase the globule size of LNVs, as depicted in **Figure S2 B(i)** and **C(i),** ostensibly owing to formation of CHL-poor and CHL-rich domains which might coalesce to form larger vesicles. Similar observations could also be deduced from the corresponding 2D-contour plots with somewhat curvilinear and nearly vertical lines (**Figure S2 A(ii), B(ii)** and **C(ii))**.

The 3D-response surface plot in **Figure S2 D(i)** depicts a distinctly nonlinear trend, attaining minimum values of PDI at the intermediate levels of HSPC and low levels of DPPC. A linear increase in the PDI of LNVs was observed with increasing levels of DPPC, whereas CHL exhibited relatively negligible effect on PDI (**Figure S2 E(i)** and **F(i))**. At higher levels of lipids, however, a decline in PDI was observed as the amounts of CHL increase from low to high levels. Intermediate levels of HSPC, low levels of DPPC and intermediate to higher levels of CHL are, therefore, found to be conducive for the formation of LNV monodispersion. **Figure S2 D(ii)** and **E(ii)** again portray the distinctly curved nature of the contour lines with the presence of a *region of minimum (i.e., nadir)* at the centre, indicating attainment of lowest magnitudes of PDI at low to intermediate levels of phospholipid and relatively higher levels of CHL. On the other hand, as shown in **Figure S2 F(ii), minimal** PDI values were obtained at the intermediate to high levels of CHL coupled with low levels of DPPC.

**Figure S2 G(i)** portrays the influence of CMPs *viz.,* DPPC and HSPC on ZP of prepared LNVs. A modest curvilinear rise in ZP values was observed with increasing levels of HSPC, while an initial rise followed by a decline was observed with rising levels of DPPC. This could be ascribable to the interaction between positively and negatively charged part of HSPC and DPPC, respectively [^2^](#_ENREF_2). Nevertheless, moderate effect of CHL on the ZP of LNVs is vividly discernible from **Figure S2 H(i)** and **I(i)**. Accordingly, the desirably high magnitudes of ZP were observed at high levels of HSPC, intermediate levels of DPPC, and intermediate to high levels of CHL. The corresponding concentric patterns in 2D-contour plots (**Figure S2 G(ii), H(ii)** and **I(ii))** conspicuously ratify analogous effects of HSPC and DPPC on ZP, indicating maximum value of ZP can be obtained at the high levels of HSPC together with intermediate levels of DPPC and CHL both.

The 3D-response surface plot in **Figure S2 J(i)** depicts the effect of HSPC and DPPC levels on EE. Somewhat linear inclining patterns of EE values were observed with rising HSPC and DPPC levels. The most desirable attribute of maximal value of EE was achieved at their corresponding highest levels, attributable to the availability of high amounts of both the lipids to entrap the drug within the phospholipid bilayers. Further, **Figures S2 K(i)** and **L(i)** too indicate a milder increment in EE values with escalating levels of either of the lipids, though a declining trend in EE is observed with increasing CHL levels from low to high. The latter effect could be attributed to tendency of CHL to compete with the drug molecules for getting incorporated into the phospholipid bilayers [^3^](#_ENREF_3). The respective 2D-contour plots, depicted in **Figure S2 J(ii), K(ii)** and **L(ii),** discern similar observations, i.e., high levels of both the lipids and low levels of CHL are favourable for attaining high values of drug EE of LNVs.

**Figure S2 M(i)** illustrates the influence of both the lipids on drug release at 8h (T8h). A somewhat linear descending trend in cumulative drug release in 8h was observed with rising levels of HSPC, while a relatively obscure relationship was noticeable with increasing DPPC levels ratifying the prevalence of interaction, *i.e.,* lack of parallelism. Maximum drug release was accordingly achieved at the lowest levels of both the lipids, HSPC and DPPC. This release retarding behaviour of phospholipids could be ascribable to their existence as gel phase at a temperature of 37°C, which is quite lower than their respective transition temperatures of 53ºC and 41ºC for HSPC and DPPC, respectively. Likewise, in **Figure S2 N(i)** and **Figure S2 O(i),** a decline in T8h was observed as the amount of HSPC and DPPC was increased from low to high levels, while the presence of CHL tended to accelerate the drug release at various levels of the phospholipids. This could be attributed to the fact that incorporation of CHL is documented to fluidize the rigid layer of saturated phospholipids and speed up the release of encapsulated drug [^4^](#_ENREF_4)^,^ [^5^](#_ENREF_5). The corresponding 2D-contour plots in **Figure S2 M(ii), N(ii)** and **O(ii)** depict analogous observations with minimal values of drug release at the higher levels of HSPC or DPPC, coupled with intermediate to high levels of CHL.

**References**

1. Chen J, Cheng D, Li J, Wang Y, Guo JX, Chen ZP, et al. Influence of lipid composition on the phase transition temperature of liposomes composed of both DPPC and HSPC. *Drug Devel Ind Pharm* 2013; **39**: 197-204.

2. Chen J, He CQ, Lin AH, Xu F, Wang F, Zhao B, et al. Brucine-loaded liposomes composed of HSPC and DPPC at different ratios: *In vitro* and *in vivo* evaluation. *Drug Devel Ind Pharm* 2014; **40**: 244-251.

3. Mahmud M, Piwoni A, Filiczak N, Janicka M, Gubernator J. Long-circulating curcumin-loaded liposome formulations with high incorporation efficiency, stability and anticancer activity towards pancreatic adenocarcinoma cell lines *in vitro*. *PloS One* 2016; **11**: e0167787

4. Dos Santos N, Mayer LD, Abraham SA, Gallagher RC, Cox KAK, Tardi PG, et al. Improved retention of idarubicin after intravenous injection obtained for cholesterol-free liposomes. *Biochim Biophys Acta* 2002; **1561**: 188-201.

5. Allen CDSN, Dos Santos N, Gallagher R, Chiu GNC, Shu Y, Li WM, et al. Controlling the physical behavior and biological performance of liposome formulations through use of surface grafted poly (ethylene glycol). *Biosci Rep* 2002; **22**: 225-250.
